# Harnessing Renewable Lignocellulosic Potential for Sustainable Wastewater Purification

**DOI:** 10.34133/research.0347

**Published:** 2024-04-04

**Authors:** Bin Wang, Jiaming Wang, Zhaohui Hu, An-Ling Zhu, Xiaojun Shen, Xuefei Cao, Jia-Long Wen, Tong-Qi Yuan

**Affiliations:** ^1^ State Key Laboratory of Efficient Production of Forest Resources, Beijing Forestry University, Beijing 100083, China.; ^2^ Beijing Key Laboratory of Lignocellulosic Chemistry, Beijing Forestry University, Beijing 100083, China.; ^3^ Hunan Nonferrous Metals Research Institute Co. Ltd., Changsha 410000, China.

## Abstract

Utilizing renewable lignocellulosic resources for wastewater remediation is crucial to achieving sustainable social development. However, the resulting by-products and the synthetic process characterized by complexity, high cost, and environmental pollution limit the further development of lignocellulose-based materials. Here, we developed a sustainable strategy that involved a new functional deep eutectic solvent (DES) to deconstruct industrial xylose residue into cellulose-rich residue with carboxyl groups, lignin with carboxyl and quaternary ammonium salt groups, and DES effluent rich in lignin fragments. Subsequently, these fractions equipped with customized functionality were used to produce efficient wastewater remediation materials in cost-effective and environmentally sound manners, namely, photocatalyst prepared by carboxyl-modified cellulose residue, biochar-based adsorbent originated from modified lignin, and flocculant synthesized by self-catalytic in situ copolymerization of residual DES effluent at room temperature. Under the no-waste principle, this strategy upgraded the whole components of waste lignocellulose into high-value-added wastewater remediation materials with excellent universality. These materials in coordination with each other can stepwise purify high-hazardous mineral processing wastewater into drinkable water, including the removal of 99.81% of suspended solids, almost all various heavy metal ions, and 97.09% chemical oxygen demand, respectively. This work provided promising solutions and blueprints for lignocellulosic resources to alleviate water shortages while also advancing the global goal of carbon neutrality.

## Introduction

**Fig. 1.**
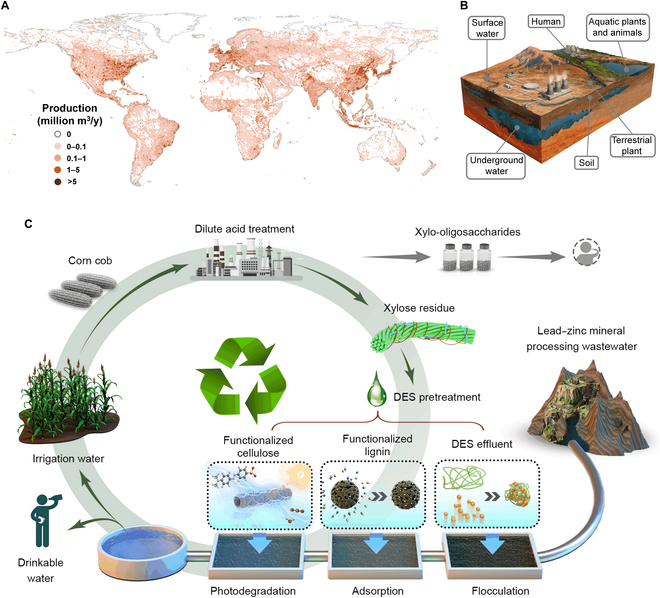
Valorization of industrial XR for relieving water crisis. (A) Gridded wastewater production at 5 arc min of spatial resolution. Data taken from [4]. (B) Schematic showing the harms of mineral processing wastewater to the ecological environment. (C) Schematic demonstrating the whole composition of XR for sequential purifying the lead–zinc mineral processing wastewater into drinkable water. First, the functional DES deconstructed XR efficiently while giving various components customized functionality. Then, 3 wastewater remediation materials were produced in simple, cost-effective, and eco-environmentally manners based on the customized structural features of various components. Finally, 3 wastewater remediation materials could sequentially purify actual lead–zinc mineral processing wastewater containing suspended solids, harmful microorganisms, heavy metal ions, and various organic pollutants, obtaining safe drinkable water.

Because of the rapid industrialization development and global population explosion, the contamination and scarcity of drinkable water resources have seriously plagued human survival and development worldwide [1]. Approximately 4.5 billion individuals currently reside in proximity to impaired water sources, and this situation is anticipated to deteriorate further in the foreseeable future (Fig. 1A) [2]. Hence, the recovery of drinkable water from wastewater is an essential way to achieve the United Nations Sustainable Development Goal 6 (Clean Water and Sanitation), which has a substantial socioeconomic impact [3,4]. A wide range of water remediation materials, such as polyacrylamide [5], polyaluminum chloride [6], graphene [7], and metal nanoparticles [8], are used in various water purification processes. However, all these materials mentioned above depend on fossil resources and have several disadvantages, such as high cost and adverse environmental toxicity [1,9,10]. Thus, a more cost-effective and sustainable strategy, such as the utilization of renewable, nonedible, and cheap lignocellulosic biomass resources for wastewater remediation, is highly desirable, which can liberate us from the dependence on fossil resources [11–13]. The value-added application of lignocellulose in the preparation of water remediation materials is an economically viable solution with a low-carbon footprint, facilitating the solution of human drinkable water problems and promoting carbon neutrality.

It is difficult to effectively convert lignocellulosic resources into high-value products due to the firmly compact structure of plant cell walls and a protective bulwark constructed by its chemical compositions [14]. The structural advantages of various components in untreated lignocellulose cannot be fully utilized, so directly preparing wastewater remediation materials usually have the characteristics of high energy consumption, low utilization rate, and inferior effect. Therefore, deconstructing lignocellulosic biomass to prepare scalable building blocks is a top priority for upgrading biomass into wastewater remediation materials, but the inherent heterogeneity of plant cell walls makes this goal elusive [15,16]. Developing a low-cost, environmental, and sustainable pretreatment technique has always been a beacon pursued by researchers [17,18]. Recently, deep eutectic solvents (DESs) have gained marked interest due to simple synthesis, recyclability, environmental friendliness, and selective solubility of biomass components [19]. More importantly, functional DES can efficiently deconstruct the lignocellulose while modifying its principal constituents to bestow targeted functionalities, thereby enhancing the economic and environmental advantages of the resulting material for wastewater remediation [20]. However, DES pretreatment also faced several challenges: (i) The incomplete regeneration of lignin fragments from the DES limits the recycling capacity of DES [21]. The ultimate disposal of DES effluents after multiple use cycles is also a cause for concern as it may lead to environmental contamination and resource waste [22]; (ii) the comprehensive utilization of all components of biomass resources under the no-waste principle is still an onerous problem [23]. These challenges above restrict the potential for attaining enduring industrial-scale implementations of DES in biomass processing [24]. Therefore, deconstructing lignocellulosic biomass and comprehensive utilization of various components to create effective materials for the collaborative elimination of diverse pollutants from wastewater not only aids in producing drinkable water but also alleviates environmental and resource strains.

**Fig. 2.**
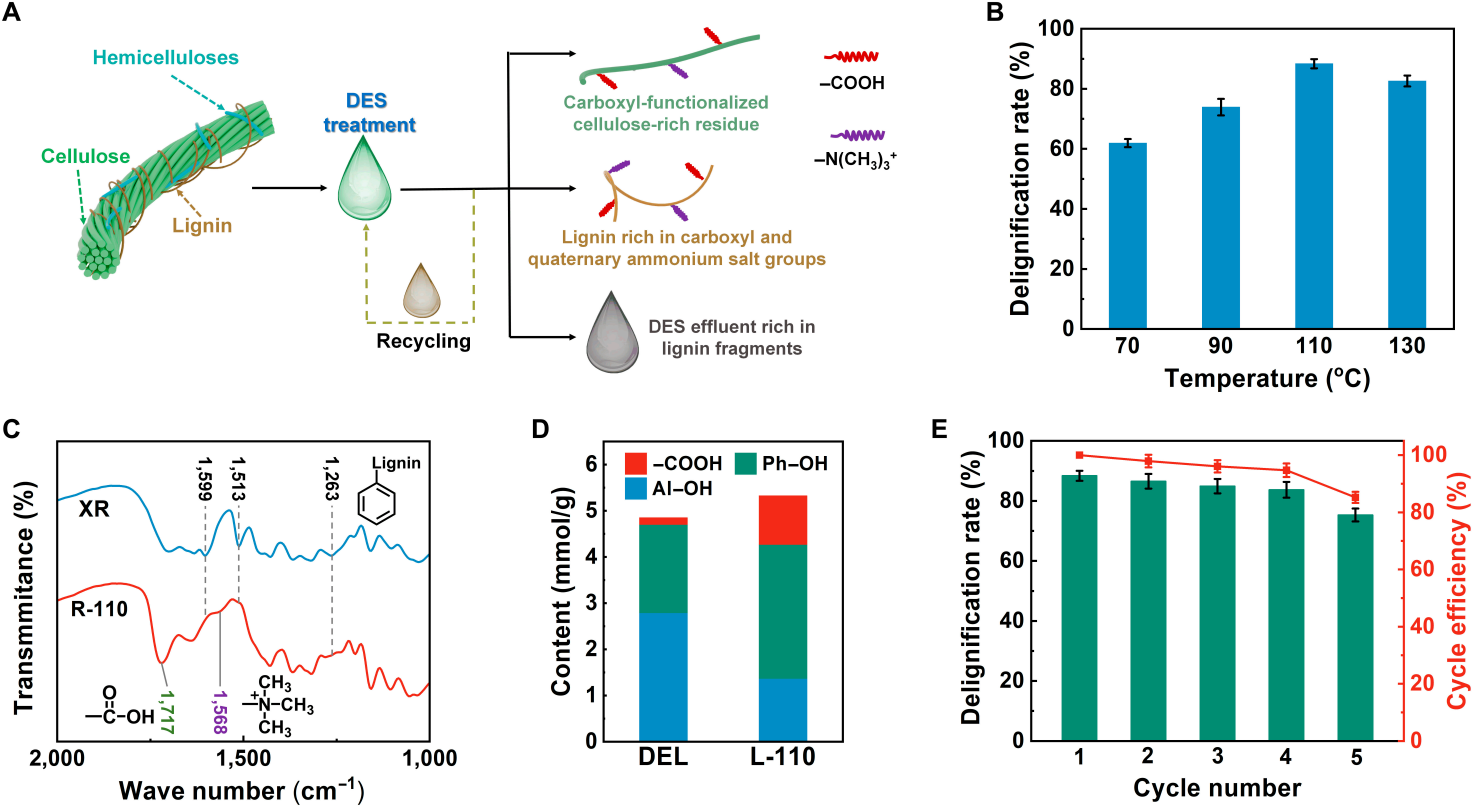
The deconstruction and functionalization effects of DES pretreatment on XR. (A) Schematic illustration of DES deconstructing XR and functionalizing the various components. (B) The delignification rate as a function of pretreatment temperature. (C) The FT-IR spectra of XR and R-110. (D) Quantitative analysis of hydroxyl and carboxyl groups in DEL and L-110 (carboxyl group, –COOH; phenolic hydroxyl group, Ph–OH; alcoholic hydroxyl group, Al–OH). (E) Cycle efficiency of DES (pretreatment temperature at 110 °C).

Herein, for the first time, we developed a cost-effective, sustainable, and holistic strategy that enables the nature of each biomass component to be unlocked for multistage industrial wastewater purification under the principle of zero waste (Fig. 1C). Wasted lignocellulose [e.g., xylose residue (XR), a by-product of the xylose industry] was chosen as raw material for the proof-of-concept demonstration due to its low cost, abundance, and underutilization. This work synthesized a novel and efficient functional DES [methyl acryloyloxyethyl trimethylammonium chloride (MTAC)/acrylic acid (AA)/aluminum chloride] for deconstructing waste lignocellulose and enhancing functionalities by introducing customized functional groups into various biomass components in situ (Fig. 2A). Subsequently, leveraging the structural features of the resulting components, a suite of wastewater remediation materials was synthesized using a simple, mild, and sustainable strategy. Biomass-based wastewater remediation materials could progressively treat suspended solids, bacteria, heavy metals, and organic pollutants in wastewater, ultimately transforming the unsuitable wastewater into potable domestic water. By utilizing functional DES to deconstruct and modify wasted lignocellulose, this study provides a systematic and feasible paradigm for producing efficient wastewater remediation materials, contributing significantly to the sustainable acquisition of drinkable water from industrial wastewater.

## Results

### Evaluation of the DES pretreatment process

In this study, XR with the characteristics of large output, cheapness, underutilization, and easy acquisition was selected as the raw material, which is considered a promising candidate for wastewater treatment. Because of the biomass recalcitrance of XR, an easy-to-operate one-pot DES pretreatment strategy was used to efficiently deconstruct XR, while endowing the main components (cellulose and lignin) with customized functionality to facilitate the subsequent preparation of wastewater remediation materials, as shown in Fig. 2A. Under optimal pretreatment conditions (110 °C), the delignification rate of XR was as high as 88.37% (Fig. 2B). In addition, cellulose-rich residue was functionalized alongside the delignification process. As shown in Fig. 2C, as compared with raw XR, the lignin-specific signal peaks (1,599, 1,513, and 1,263 cm^−1^) were significantly attenuated in the Fourier transform infrared (FT-IR) spectrum of R-110 [25], confirming the high delignification rate. Notably, the C═O stretching vibration peak at the carboxyl group (1,717 cm^−1^) was significantly enhanced, indicating the substantial introduction of carboxyl groups onto the cellulose [26]. The enhanced electrostatic repulsion and hydrophilicity caused by surface carboxyl groups would facilitate the preparation of lignin-containing cellulose nanofiber (LCNF), which was an excellent in situ carbon doping source and biological template for the preparation of carbon-nitride-based photocatalysts.

To investigate the change and functionalization of lignin structure during DES pretreatment, we used double enzymatic lignin (DEL) extracted from the identical XR as a model native lignin in XR. It could be seen from the analysis results of FT-IR (Fig. S3A) and ^1^H nuclear magnetic resonance (Fig. S3B) that a large number of carboxyl and quaternary ammonium salt groups in DES were reacted with the lignin skeleton during the delignification process. The quantitative ^31^P nuclear magnetic resonance results (Fig. 2D and Table S2) showed that the carboxyl groups of L-110 were as high as 1.06 mmol/g, which was 7 times the carboxyl group content of DEL. It is well known that abundant carboxyl groups play a vital role in promoting the adsorption capacity of lignin for cationic pollutants such as heavy metal ions [27]. In addition, a large number of quaternary ammonium salt groups on lignin would produce a mass of gas during low-temperature calcination, which provided a guarantee for the green preparation of porous biochar.

Although DESs provide a cost advantage over ionic liquids, the industrial application in effluent faces hurdles. Here, we aimed to reduce DES production costs by exploring DES recycling. The pretreatment capacity of DES gradually decreased with the increase in the cycle number due to the challenge of isolating the lignin fragments from DES and the inevitable loss of AA until the delignification rate dropped below 80% in the fifth cycle (Fig. 2E). In the industrial sector, the disposal of DES effluent would significantly increase economic and environmental burdens. Nevertheless, DES amassed a substantial concentration of lignin fragments after multiple cycles, which provided a priori condition for the establishment of a self-catalytic in situ copolymerization system. A detailed analysis of the DES pretreatment evaluation was presented in Note S10.

### Wastewater purification

This work focused on the representative lead–zinc mineral processing wastewater with high turbidity and highly hazardous characteristics as the research subject. Successfully addressing the treatment of lead–zinc mineral processing wastewater can provide a solid technical and experiential foundation for treating other types of wastewater (Fig. 1B). Moreover, we validated the versatility of various wastewater remediation materials by simulating various internal and external influencing factors that may be encountered in practice.

### Flocculation

**Fig. 3.**
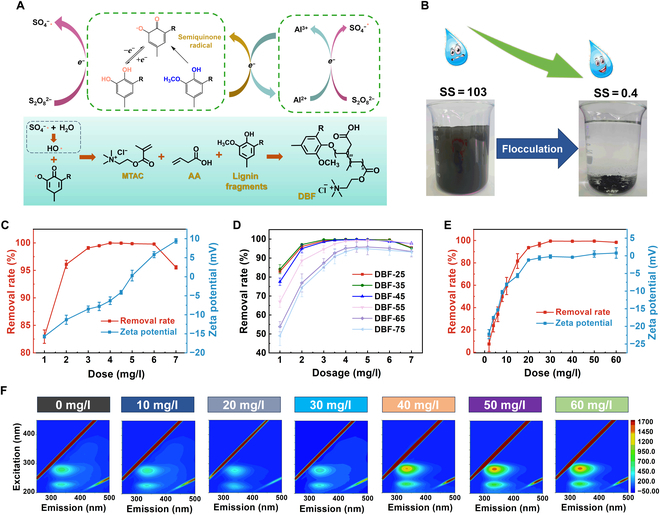
Synthesis, characterization, and properties of DES-based flocculant (DBF). (A) Schematic illustration of a lignin fragment-Al^3+^ self-catalytic system triggering the synthesis of DBF. (B) Comparison of lead–zinc mineral processing wastewater before and after flocculation (DBF-25, 5 mg/l). SS, suspended solid. (C) Removal rate and zeta potential of kaolin suspension as functions of DBF-25 dosage. (D) Removal rate of kaolin suspension by DBFs prepared at different temperatures (dosage, 5 mg/l). (E) Removal rate and zeta potential of *E. coli* suspension as functions of DBF-25 dosage. (F) 3-dimensional fluorescence spectrum spectra of the supernatant as functions of DBF-25 dosage.

Traditional organic flocculants encounter a significant hurdle related to the high costs associated with raw materials and preparation processes [11,28]. In this study, pioneering using DES effluent with severely reduced pretreatment capacity after multiple cycles as raw materials, a self-catalytic in situ copolymerization system was established on the basis of its unique characteristics, preparing a multifunctional and high-performance DES-based flocculant at ambient temperature and pressure. As illustrated in Fig. 3A, a self-catalytic system was composed of lignin fragments with reductibility and aluminum ions with redox through chelation. Within this self-catalytic system, lignin fragments with reducing groups (methoxy and phenolic hydroxyl groups) were converted into semiquinone/catechol structures with prominent redox activity [29], and the ammonium persulfate (APS) was induced to produce sulfate radicals [30]. Subsequently, the DES-based flocculant (DBF-25) was synthesized via the copolymerization of AA, MTAC, and lignin fragments. Notably, compared with traditional industrial lignin, the lignin fragments in this study have excellent water solubility, outstanding reaction accessibility, and high reactivity due to the sufficient degradation of lignin during the DES pretreatment process, which provides a solid foundation for the efficient operation of the self-catalytic in situ copolymerization.

As described in Note S11, structural analyses showed that the self-catalytic in situ copolymerization was successfully performed, preparing DBF-25 at ambient temperature and pressure. The lignin fragments could not only establish a self-catalytic system with Al^3+^ but also participate in the copolymerization reactions with AA and MTAC. DBF-25 had a high positive zeta potential and a molecular weight of up to 139,940 g/mol (Fig. S5A and C), which were conducive to charge attraction and bridging effect during the flocculation process, respectively [11]. After a series of preliminary experiments, this study determined the use of DBF-25 (5 mg/l) to flocculate the actual lead–zinc mineral processing wastewater. As shown in Fig. 3B, the turbidity of lead–zinc mineral processing wastewater was rapidly reduced from 103 to 0.4 after flocculation. To validate the universality of DBF-25 and explore the flocculation mechanism, we systematically conducted a series of flocculation tests using kaolin suspension to simulate wastewater. In the initial stage of increasing dosage, the addition of positively charged DBF-25 reduced the electrostatic repulsion between negatively charged kaolin particles due to charge neutralization, and the removal rate increased rapidly with the cooperation of the bridging effect (Fig. 3C). When the dosage of DBF-25 reached 4 mg/l, the optimal dosage was reached, and the removal rate of kaolin exceeded 99%. Notably, at this point, the zeta potential of the supernatant was −6.3, which indicated the existence of a charge patching effect. Upon further dosage increase beyond the optimum, a phenomenon of antiflocculation emerged, where the zeta potential continued to increase and the removal rate decreased. This was because kaolin particles adsorbed excessive flocculants with a positive charge, increasing electrostatic repulsion among particles and reducing flocculation efficiency. A detailed discussion on flocculation tests is described in Note S12.

The internal factors of DES-based flocculants were discussed by preparing a series of flocculants at different temperatures. Structural analysis showed that AA primarily participated in the copolymerization reaction at higher temperatures, while lower temperatures favored the copolymerization of MTAC (Note S11). As shown in Fig. 3D, DBF-35, with the highest molecular weight and positive charge density, demonstrated the best flocculation performance, slightly superior to DBF-25. However, considering the cost escalation and the increased operational complexity resulting from heating, DBF-25 was regarded as the optimal solution. For external factors (Note S12), DBF-25 demonstrated robust resistance to wastewater pH and coexisting ions, making it suitable for a wide range of scenarios.

Actual wastewater often contains a large number of harmful microorganisms, which can cause various diseases [31]. However, current commercial disinfectants can react with organic pollutants in wastewater, causing secondary pollution [32]. Therefore, the development of a dual-functional flocculant with flocculation and sterilization performances not only reduces the difficulty and cost of wastewater purification but also enhances environmental benefits [33]. In this study, the sterilization performance of DBF-25 was evaluated using representative *Escherichia coli* as a research case. The results showed that at the optimal dosage of 30 mg/l, DBF-25 achieved a removal rate of 99.5% for the *E. coli* suspension (Fig. 3E). Interestingly, as the dosage continued to increase, the supernatant’s removal rate and zeta potential remained stable without showing antiflocculation behavior. Through the 3-dimensional fluorescence spectrum of the supernatant shown in Fig. 3F, significantly enhanced signals belonging to soluble products (200 to 240/280 to 380 nm) and internal aromatic proteins (250 to 300/300 to 400 nm) of *E. coli* were detected in the supernatant [33]. In general, these signals could be detected in small amounts due to the metabolism of *E. coli* [34]. However, these signals were detected to be significantly enhanced when the dosage of DBF-25 exceeded 30 mg/l, indicating that the *E. coli* cell structure was disrupted. Moreover, the intensities of these signals increased with increasing dosages of DBF-25. These phenomena illustrated that when DBF-25 purified *E. coli* suspension, it was mainly used for flocculation when the dosage was small, but when there was enough DBF-25, the sterilization effect could be clearly activated. The sterilization ability of DBF-25 was due to DBF-25 firmly adsorbing to the surface of *E. coli* through charge attraction, impeding normal cellular activities [35]. Moreover, the abundant quaternary ammonium salt groups on DBF-25 induced the denaturation of cellular proteins on the cell membrane, leading to the killing of *E. coli* and the rupture of cell membranes. The discussion on the flocculation and sterilization of *E. coli* suspension by DBF-25 is shown in Note S13.

In summary, this section used DES effluent as the precursor and required only minute amounts of APS initiator to establish a self-catalytic in situ copolymerization system, achieving a ground-breaking synthesis of DBF-25 under ambient aqueous conditions. Moreover, DBF-25 exhibited fascinating dual functionality, flocculation, and sterilization and had outstanding universality. From feedstock to synthesis procedures and application efficacy, the “waste treating waste” strategy embodied in this study exemplified remarkable sustainability on all fronts.

### Adsorption

**Fig. 4.**
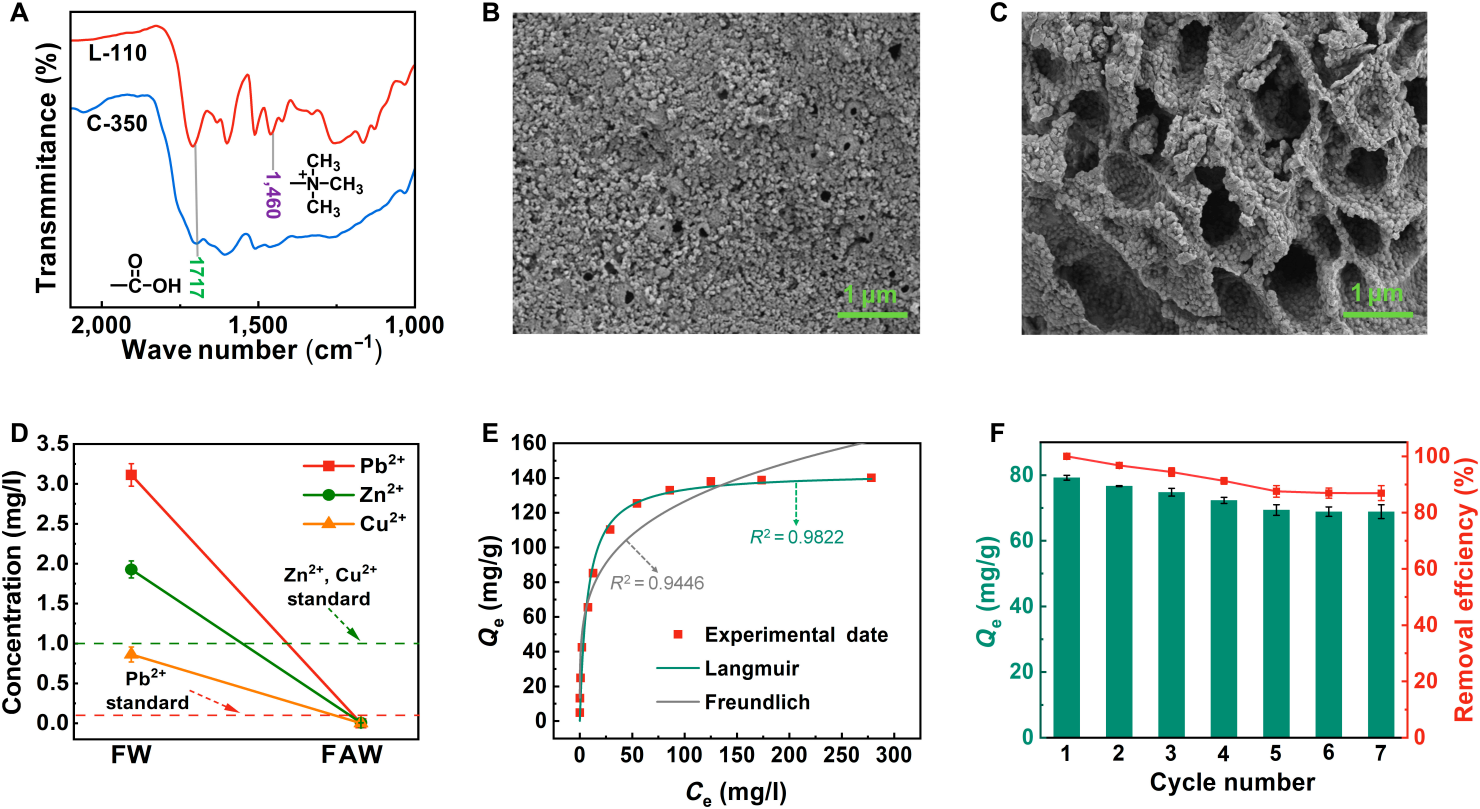
Characterization and properties of lignin-based biochar. (A) The FT-IR spectra of L-110 and C-350. (B) Scanning electron microscopy image of L-110. (C) Scanning electron microscopy image of C-350. (D) Concentration of heavy metals in lead–zinc mineral processing wastewater before and after adsorption (C-350, 0.5 g/l). (E) Adsorption isotherms for Pb^2+^ on C-350 and the corresponding isothermal adsorption models (C-350, 0.5 g/l). (F) Adsorption capacity of C-350 at different regeneration cycles [initial Pb^2+^ concentration, 35 and 0.5 g/l (C-350)].

Because of the high carbon content, unique aromatic structure and rich oxygen-containing functional group, lignin is considered to be a favorable competitor for preparing carbon materials to adsorb heavy metals. However, the preparation of lignin-based carbon materials usually requires the use of large amounts of activators, strict high-temperature conditions, and long pyrolysis times, resulting in high cost and low yields [36,37]. Although biochar production is characterized by relatively mild preparation conditions and high yields compared to other carbon materials, it is regrettable that the vast majority of lignin-based biochar exhibits extremely low specific surface areas (potentially subpar to the raw lignin) and a scarcity of adsorption functional groups due to the inherent structural limitations of industrial lignin [38,39]. Against this backdrop, the present study used DES to simultaneously deconstruct XR and functionalize lignin, obtaining lignin with tailored structures. L-110 was rich in carboxyl and quaternary ammonium salt groups. By using L-110 as the precursor, without the need for additional activation agents or modifying reagents, the lignin-based biochar C-350 with a high yield of 67.78% was obtained at low-temperature annealing of 350 °C (Fig. S7B). From the analysis results of Note S14, during the low-temperature calcination process, C-350 retained abundant carboxyl groups (Fig. 4A) and significantly increased the specific surface area through the large amount of gas generated by the pyrolysis of the quaternary ammonium salt (Fig. S7A). After calcination, the flat surface of L-110 changed to the porous structure of C-350 (Fig. 4B and C). The rich carboxyl groups and porous structure provide innate conditions for C-350 to adsorb heavy metal ions efficiently.

Utilizing C-350 (0.5 g/l), the study conducted adsorption treatment on the flocculated lead-zinc mineral processing wastewater (FW) to remove heavy metal ions primarily. As illustrated in Fig. 4D, the concentrations of various heavy metal ions in the FAW (FW after adsorption treatment) were greatly reduced, reaching below the drinkable water standard. To investigate the adsorption mechanism, universality, and cycle performance of C-350 for heavy metals, a series of adsorption tests were conducted on simulated heavy-metal wastewaters. As discussed in Note S15, the adsorption process of Pb^2+^ by C-350 conformed to the Langmuir isotherm model (Fig. 4E), which belonged to monolayer adsorption [40]. The theoretical maximum adsorption capacity was as high as 143.21 mg/g (Table S3), which was superior to the majority of biochars reported in the literatures [38–40]. In addition, the kinetic fitting results showed that the adsorption process exhibited high fitting data for both pseudo-first-order and pseudo-second-order models (Fig. S9A), involving both physisorption and chemisorption [41]. Furthermore, C-350 maintained more than 86% adsorption capacity after 7 cycles, demonstrating excellent recycling capacity (Fig. 4F).

The annealing temperature had a decisive impact on the functional group composition, specific surface area, and yield of biochar [42]. Brief Note S14 analysis results, the yield and carboxyl content of biochar decreased with increasing annealing temperature, especially when the annealing temperature exceeded 500 °C, and almost no carboxyl groups were retained (Fig. S7B and C). Moreover, an appropriate increase in annealing temperature helped to increase the specific surface area of lignin-based biochar. When the annealing temperature exceeded 350 °C, the specific surface area decreased with the increase in annealing temperature due to insufficient gas supply from the pyrolysis of the quaternary ammonium salt groups (Fig. S7A). Adsorption test results indicated that C-350 exhibited the strongest adsorption capacity for Pb^2+^, and C-350 was regarded as the optimal choice (Fig. S7B). For external factors, C-350 exhibited commercially acceptable resistances to wastewater pH and ionic strength, which was specifically discussed in Note S15. In addition, for Zn^2+^- and Cu^2+^-simulated wastewater, C-350 showed the maximum adsorption capacities of 110.64 and 64.84 mg/l, respectively (Table S3). Moreover, Fig. S10B showed that C-350 could effectively remove 3 metal ions for the Pb^2+^–Zn^2+^–Cu^2+^ mixed wastewater at the same time. These simulation results indicated that C-350 held great promise for the purification of complex aquatic environments in actual wastewater treatment scenarios.

Overall, lignin with carboxyl and quaternary ammonium functional groups was obtained via novel DES pretreatment of XR. The efficient 350 °C could be prepared at temperatures as low as 350 °C without additional activators or modified reagents. Furthermore, 350 °C exhibited outstanding adsorption capabilities for heavy metals under various ionic strength and pH conditions.

### Photocatalytic degradation

**Fig. 5.**
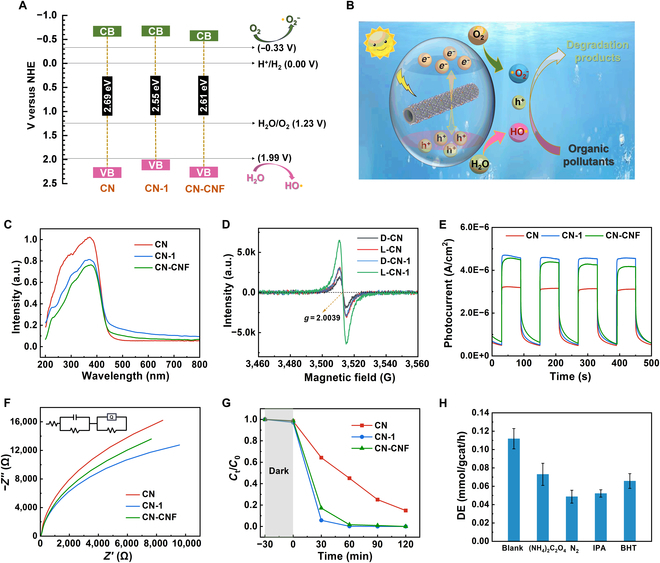
Characterization and properties of carbon nitride doped with LCNF. (A) Bandgap alignment of CN, CN-1, and CN-CNF. NHE, normal hydrogen electrode. (B) Schematic illustration of organic pollutant photodegradation by CN-1 under 300-W xenon lamp simulated sunlight (2.5 kW/m^2^). (C) Ultraviolet-visible absorbance spectra of CN, CN-1, and CN-CNF. a.u., arbitrary units. (D) In situ EPR signals of CN and CN-1 [dark (D), 10 min; light (L), 10 min; under 300-W xenon lamp simulated sunlight). (E) Photocurrent response of CN, CN-1, and CN-CNF. (F) Electrochemical impedance spectra of CN, CN-1, and CN-CNF. (G) Photodegradation of MO by CN, CN-1, and CN-CNF (MO concentration, 10 mg/l; CN-1 concentration, 0.5 mg/ml; 300-W xenon lamp simulation sunlight). (H) Effect of various scavengers on the photocatalytic activity of CN-1 toward the degradation efficiency (DE) of MO. The photodegradation ability of CN-1 to MO decreased severely under the N_2_ atmosphere (•O_2_^−^), followed by the addition of isopropanol (IPA; a scavenger for •OH). The addition of butylated hydroxytoluene (BHT, a scavenger for •R) had a weak effect on the photodegradation ability of CN-1.

In general, after traditional wastewater treatment processes such as flocculation and adsorption, trace amounts of organic pollutants often persist in the wastewater in a stable form, making them difficult to degrade [43]. Photocatalytic degradation, as a recently popular advanced oxidation technology, possesses advantages such as broad-spectrum activity, low energy consumption, and minimal secondary pollution, showing promising applications [44–46]. In this study, an in situ carbon doping strategy was applied to improve the photodegradation ability of graphitic carbon nitride for organic pollutants. The resulting cellulose residue was rich in carboxyl groups, which made it easy to obtain LCNF through physical homogenization only [26]. The abundant carboxyl groups and nanotube structure of LCNF enhanced urea’s adsorption, thereby improving carbon doping’s efficiency. The carbon-doped g-C_3_N_4_ (CN-1) was prepared by in situ thermal condensation of a urea mixture with an equivalent LCNF suspension mass (1 wt%). Through structural analyses, it could be seen that the introduction of LCNF enhanced the light absorption capacity (Fig. 5C) and increased the specific surface area (Fig. S12). Under the premise of ensuring the generation of superoxide radicals (•O_2_^−^) and hydroxyl radicals (•OH), CN-1 exhibited the narrowest bandgap. Moreover, CN-1 exhibited a weaker photoluminescence (PL) signal (Fig. S14B), a stronger electron paramagnetic resonance (EPR) signal attributed to the π-conjugated aromatic delocalized structures (Fig. 5D and Fig. S14A), higher photocurrent response (Fig. 5E), and lower interface electron transfer resistance (Fig. 5F). These results indicated that the introduction of LCNF enhanced the light absorption capacity, reduced the recombination of photogenerated electron–hole pairs, and promoted the migration of photogenerated carriers to the catalyst surface, thereby enhancing the photocatalytic performance of CN-1. These detailed discussions were presented in Note S16.

After consecutive flocculation and adsorption, FAW still contained trace organic pollutants, with a chemical oxygen demand (COD) of 77 mg/l. A 300-W xenon lamp simulating sunlight (2.5 kW/m^2^) was used as the light source, and FAW was subjected to photodegradation treatment for 4 h with CN-1 (0.5 mg/ml). After photodegradation treatment of FAW (FAPW), the COD of wastewater dropped to 3 mg/l, which was below the drinkable water standard of 5 mg/l (Table S4). Subsequently, the universality and photodegradation mechanism of CN-1 was discussed by using methyl orange (MO) solution as the simulated wastewater. As described in Note S17, CN-1 exhibited a superior photodegradability to MO than CN and CN-CNF and CN-1 (0.5 mg/ml) could almost completely photodegrade MO (10 mg/l) within 60 min (Fig. 5G). Observingly, CN-1 exhibited stronger photocatalytic performance than CN-CNF, primarily attributed to the highly condensed lignin with excellent light-absorbing ability contained in LCNF.

For internal factors (the LCNF added amount), summarizing the analysis results in Note S16, CN-1 was considered the optimal solution. The introduction of excess LCNF made the bandgap too narrow to be excited to generate •OH or even •O_2_^−^ (Figs. S15 to S17) and made the photoelectric performance stronger (Fig. S18). As for external factors, an appropriate increase in CN-1 dosage could accelerate the photodegradation of MO, but too much CN-1 would hinder light absorption, making it impossible to continue to increase the photodegradation rate (Fig. S21). In addition, CN-1 showed excellent photodegradation effects on methylene blue, naproxen, bisphenol A, and acyclovir, demonstrating the outstanding universality (Fig. S22), indicating the outstanding universality of CN-1 for the degradation of organic pollutants. As mentioned above, CN-1 could theoretically guide the generation of •O_2_^−^ and •OH, which was confirmed by EPR detection results (Fig. S23). Moreover, various shielding tests for photogenerated holes and free radicals were performed. As shown in Fig. 5H, the •O_2_^−^ and •OH played critical roles in MO degradation, followed by the alkyl radicals (•R). Furthermore, the holes with a high oxidation potential could directly participate in MO degradation [27].

### Compilation and evaluation

**Fig. 6.**
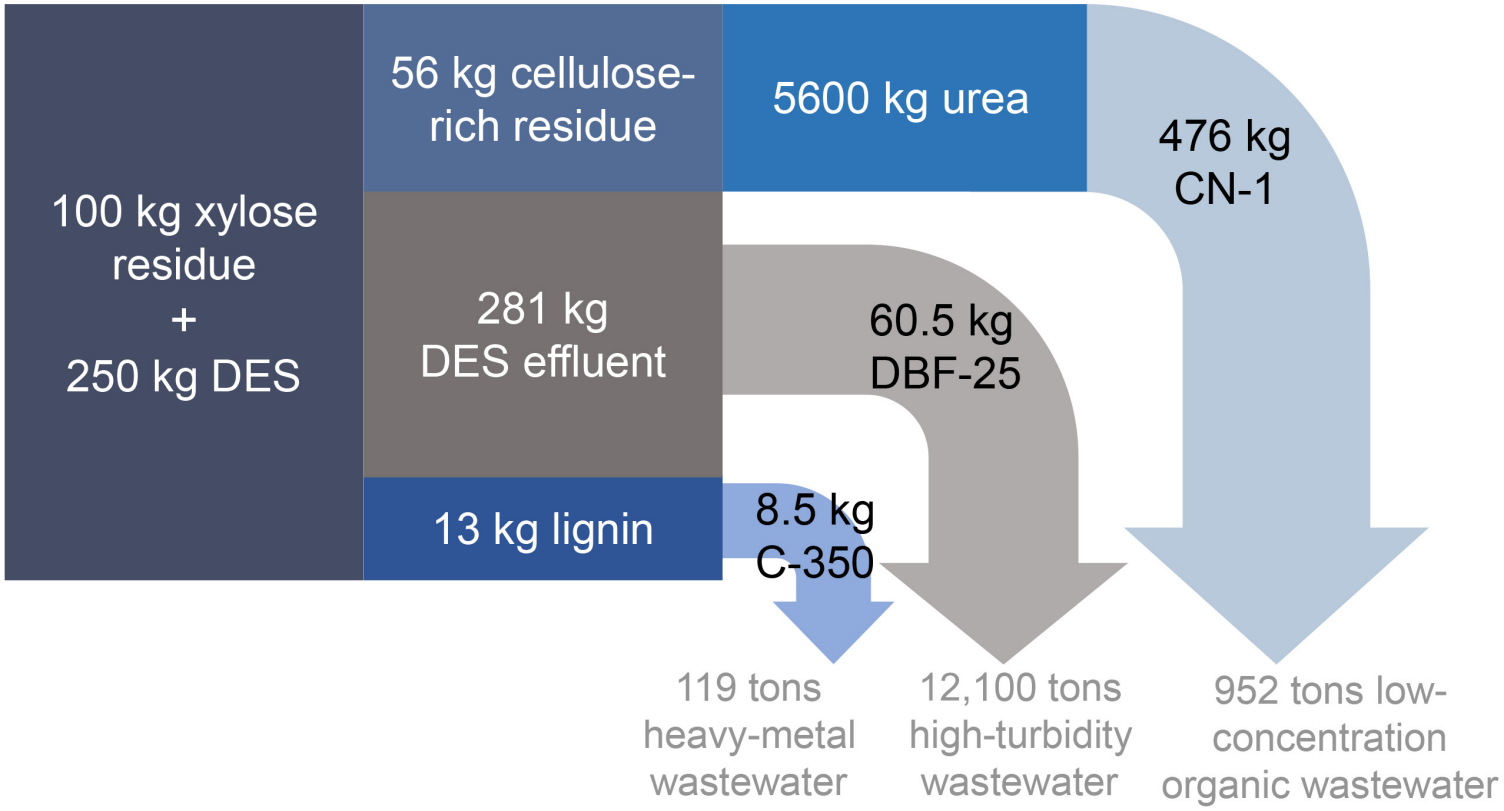
Mass balance of the sustainability strategy in this study. DES was recycled 4 times under optimal pretreatment conditions (solid–liquid ratio, 1:10; pretreatment temperature, 110 °C; time, 3 h). The dosages of biochar C-350, flocculant DBF-25, and photocatalyst CN-1 were 0.5, 0.005, and 0.5 g/l, respectively. Among them, the number of efficient recycling of C-350 is 7. A detailed description is shown in Fig. S27.

After 3 sequential purification steps, the lead–zinc mineral processing wastewater met the strictest limits of mainstream standards for drinking water (Fig. S25 and Table S4). The universalities of these wastewater remediation materials have been verified through the above series of simulated purification tests. In this context, the mass balance assessment was carried out on the basis of 100 kg of XR as raw material. Theoretically, not only for the raw material XR but also for the DES, the whole components could be applied to prepare various wastewater remediation materials (Fig. 6). Under the corresponding optimal preparation conditions, 476 kg of photocatalyst CN-1, 8.5 kg of biochar adsorbent C-350, and 60.5 kg of DES-based flocculant DBF-25 are synthesized, which can purify 12,100, 119, and 952 tons of corresponding types of wastewaters, respectively.

Taking the adsorption process with the lowest purification capacity as the standard, 100 kg of XR could sequentially purify at least 119 tons of wastewater into drinkable water. For China alone, the annual output of XR is 40 million tons [45]. It is roughly estimated that 4.76 × 10^10^ tons of wastewater can be purified into drinkable water, which exceeds 74% of China’s total annual wastewater discharge (6.39 × 10^10^ tons, including industrial wastewater and domestic sewage) (https://www.mee.gov.cn/hjzl/sthjzk/zghjzkgb/). Moreover, this strategy is not limited to XR but has foreseeable applicability to other lignocellulosic biomass, which allows locally appropriate adjustments to be made on the basis of the distribution of lignocellulosic biomass, facilitating the industrialization process.

## Discussion

Briefly, we successfully developed a new sustainable strategy to sequential purify wastewater into drinkable water using the whole composition of industrial by-product XR. This work efficiently deconstructed the main components of XR while endowing them with customized functionalization, and then produced 3 wastewater remediation materials based on the distinctive structural features of these components, including flocculants, adsorbents, and photocatalysts. In contrast to other corresponding material preparation methods, these production processes in this study avoid the use of a large number of harmful reagents, high temperature, sophisticated equipment, and lengthy processing times, demonstrating promising environmental and economic benefits. In terms of effects, the 3 materials not only demonstrate superior purification capabilities but also have outstanding universality. In particular, these 3 materials have quite compatible and complementary properties with each other and can carry out sequential purification of most actual wastewater. Taking the mineral processing wastewater with high hazard and difficult-to-treat characteristics as an example, the 3 materials successfully purified the mineral processing wastewater step by step into safe and drinkable water. It is roughly estimated that the annual output of XR in China alone can purify approximately 74% of the nation’s total yearly wastewater discharge for consumption. This study contributes solidly to the global-water-renewable resource nexus and provides promising solutions and blueprints for alleviating ever-mounting challenges posed by the water crisis and resource scarcity while achieving sustainable development.

## Materials and Methods

### Materials and chemicals

The industrial XR was obtained by the hemicelluloses hydrolysis of corn cob into xylo-oligosaccharides, which was provided by Shandong Longlive Bio-technology Co. Ltd., China. The production process of industrial XR is as follows: The crushed corn cob as the raw material was pretreated under acidic conditions (diluted sulfuric acid) at 110 °C for 0.5 h and then hydrolyzed at 120 °C for 2.0 h. The solid generated after hydrolysis is the industrial XR, and its chemical compositions and original picture are shown in Table S1 and Fig. S28, respectively. AA (99%), MTAC (80 wt% in H_2_O), aluminum chloride (99%), and other chemicals were purchased from Aladdin Reagent Co. Ltd. All the chemicals are analytical purity and have not been further processed. Tempo-oxidized nanocellulose (CNF) and lead–zinc mineral processing wastewater were donated by Tianjin Woodelf Biotechnology Co. Ltd. and Hunan Research Institute for Nonferrous Metals Co. Ltd., respectively.

### DES preparation and pretreatment

DES was obtained by mixing AA, MTAC and aluminum chloride at the molar ratio of 1:1:0.05 and stirring at 70 °C to a transparent solvent. The mass ratio of XR and DES was 1:10 mixed and heated at different temperatures (70, 90, 110, and 130 °C, respectively) for 3 h. After cooling, 3 times the volume of ethanol was added. Subsequently, cellulose-rich residues were obtained by solid–liquid separation and freeze-dried, named R-70 to R-130, respectively. The ethanol in the obtained supernatant was removed by vacuum distillation and recycled. Next, the lignin components (named L-70 to L-130, respectively) were precipitated by adding 3 volumes of deionized water to the distilled DES liquor. The supernatant was further dehydrated by vacuum distillation to obtain regenerated DES. The regenerated DES was reused according to the above steps until the pretreatment effect was seriously decreased (the delignification rate was less than 80%), and the DES effluent rich in lignin fragments was obtained.

### Wastewater remediation materials preparation and performance test

#### Flocculant preparation and flocculation test

DES-based flocculant was prepared by in situ self-catalytic copolymerization using DES effluent as raw material. Specifically, 5 g of the DES effluent and 2.5 g of deionized water mixture were added to the flask for mixing, and N_2_ was passed for 20 min to remove O_2_. Subsequently, APS with a mass fraction of 0.03% of the DES effluent was injected to establish the self-catalytic copolymerization system. Under an N_2_ atmosphere, the mixture was reacted at different temperatures (25, 35, 45, 55, 65, and 75 °C) for 4 h. Finally, the reacted mixture was dropped into ethanol solution to precipitate DES-based flocculants (named DBF-25 to DBF-75).

After a series of preliminary experiments, it was determined that DBF-25 (5 mg/l) was used to flocculate the actual lead–zinc mineral processing wastewater, and the turbidity before and after flocculation was tested to measure the flocculation effect. The remaining flocculation and sterilization tests using kaolin suspension and *E. coli* suspension as simulated wastewaters were described in detail in Notes S1 and S2, respectively.

#### Adsorbent preparation and adsorption test

L-110 was used as a precursor and calcined in a vacuum tube furnace under an Ar atmosphere at different temperatures (250, 300, 350, 400, 500, 600, and 700 °C) for 60 min to prepare a series of lignin-based biochars, which were named from C-250 to C-700. The obtained biochar was ground, washed with water, and dried in an oven at 60 °C.

The FW was adsorbed with C-350 (0.5 g/l). The adsorption effect was measured by detecting the concentration of heavy metals (Pb^2+^, Zn^2+^, and Cu^2+^) in the wastewater before and after adsorption. The remaining adsorption tests were described in Note S3.

#### Photocatalyst preparation and photodegradation test

LCNF was prepared by homogenizing the cellulose-rich residue using a high-pressure homogenizer (Microfluidizer M-110EH-30, MFIC, USA), and its concentration was adjusted to 1 wt%. Subsequently, different mass ratios of LCNF suspension and urea (0.5, 1, 2.5, 5, 10, 50:1) were mixed and sonicated for 20 min. The mixture was then transferred to a corundum crucible and sealed with tinfoil. The thermal condensation process was performed in a muffle furnace at 500 °C for 2 h, and a series of carbon-doped graphitic carbon nitrides (named CN-0.5 to CN-50) were obtained. In addition, CN-CNF was synthesized by calcination of 1.0 wt% of CNF suspension with a urea mass ratio of 1:1 at 500 °C for 2 h, while graphite carbon nitride CN was prepared by calcining of urea in a muffle furnace.

For lead–zinc mineral processing wastewater treated successively by flocculation and adsorption (FAW), photodegradation was carried out with CN-1 (0.5 g/l) under 300-W xenon lamp simulated sunlight (2.5 kW/m^2^) for 4 h. The mineralization effect was judged by detecting the change of COD before and after photodegradation. The remaining photodegradation tests were described in Note S4.

## Data Availability

The data that support the findings of this study are available from the corresponding author upon reasonable request.
