# Supplementary material for: Harnessing Renewable Lignocellulosic Potential for Sustainable Wastewater Purification
Source: Research (Wash D C). 2024 Apr 4;7:0347. doi: 10.34133/research.0347 (PMC10993153; doi:10.34133/research.0347)
Supplement: Supplementary 1 — Figs. S1 to S28 Tables S1 to S4 [file research.0347.f1.docx]

Supporting Information

Title

Harnessing Renewable Lignocellulosic Potential for Sustainable Wastewater Purification

**Authors**

Bin Wang^1,2^, Jiaming Wang^1,2^, Zhaohui Hu^1,2^, An-Ling Zhu^3^, Xiaojun Shen^1,2*^, Xuefei Cao^1,2^, Jia-Long Wen^1,2^, Tong-Qi Yuan^1,2*^

**Affiliations**

^1^ State Key Laboratory of Efficient Production of Forest Resources, Beijing Forestry University, Beijing 100083, China.

^2^ Beijing Key Laboratory of Lignocellulosic Chemistry, Beijing Forestry University, Beijing 100083, China.

^3^ Hunan Nonferrous Metals Research Institute Co., Ltd, Changsha 410000, China.

^*^Address correspondence to: shenxiaojun@bjfu.edu.cn (Xiaojun Shen) and ytq581234@bjfu.edu.cn (Tong-Qi Yuan)

**Contents**

**Methods**

**Note S1.** Preparation of double enzymatic lignin (DEL).

**Note S2.** Flocculation for kaolin suspension.

**Note S3.** Flocculation and sterilisation for *E. coli* suspension.

**Note S4.** Adsorption test.

**Note S5.** Photodegradation test.

**Note S6.** The chemical compositions of the cellulose-rich substrates.

**Note S7.** The equations for pretreatment performance analysis.

**Note S8.** Characterisation methods.

**Note S9.** Photoelectrochemical measurement.

**Results**

**Note S10.** Evaluation of DES pretreatment process.

**Note S11.** Characterisation analysis for DES-based flocculant.

**Note S12.** Flocculation evaluation for kaolin suspension.

**Note S13.** Flocculation and sterilisation evaluation for *E. coli* suspension.

**Note S14.** Characterisation analysis for lignin-based biochar.

**Note S15.** Adsorption evaluation for heavy metals.

**Note S16.** Characterisation analysis for photocatalyst.

**Note S17.** Photodegradation evaluation for organic pollutants.

**Fig. S1** 2D-HSQC spectra of the lignin samples.

**Fig. S2** ^31^P NMR spectra of the lignin samples.

**Fig. S3** FT-IR and ^1^H NMR spectra of lignin samples.

**Fig. S4** FT-IR and ^1^H NMR spectra of flocculants.

**Fig. S5** Structural characterisation of DES-based flocculants.

**Fig. S6** The influence of external factors on the flocculation performance.

**Fig. S7** Structural characterisation of lignin-based biochar samples.

**Fig. S8** SEM images and the corresponding elemental mapping analyses of C-350 and C-350-Pb.

**Fig. S9** The influence of external factors on the adsorption capacity.

**Fig. S10** The adsorption capacity for other heavy metal ions.

**Fig. S11** XPS high-resolution spectra of catalysts.

**Fig. S12** Micromorphology and BET results of catalysts.

**Fig. S13** Bandgap and VB-XPS calculations.

**Fig. S14** EPR and PL spectra.

**Fig. S15** Bandgap calculation of photocatalyst samples with different LCNF content.

**Fig. S16** VB-XPS calculation of photocatalyst samples with different LCNF content.

**Fig. S17** Schematic diagram for bandgap alignment of photocatalyst samples with different LCNF content.

**Fig. S18** Photochemistry spectra of photocatalyst samples with different LCNF content.

**Fig. S19** Degradation efficiency (DE) of MO by CN-1 and CN-CNF.

**Fig. S20** Photodegradation of MO by photocatalyst samples with different LCNF content.

**Fig. S21** Photodegradation of MO by CN-1 with different dosages.

**Fig. S22** Photodegradation of other organic pollutants.

**Fig. S23** EPR spectra of CN-1.

**Fig. S24** Free radical scavenging tests.

**Fig. S25** The water quality test report of purified mineral processing wastewater.

**Fig. S26** Schematic illustration of lead-zinc mineral processing wastewater being purified into drinkable water.

**Fig. S27** Mass balance of the strategy in this study.

**Fig. S28** The original picture of industrial XR.

**Table S1** Solid yield and chemical compositions of substrates after different pretreatment conditions (%).

**Table S2** Quantification of lignin component by quantitative ^31^P NMR spectroscopy (mmol/g) and the molecular weights of lignin samples (g/mol).

**Table S3** The isothermal adsorption model parameters of C-350.

**Table S4** Results of various parameters in wastewater and the strictest drinking water standards.

**Supplementary References 1-39**

**1. Methods**

**Note S1.** **Preparation of double enzymatic lignin (DEL).**

The XR (5.0 g) was mixed with the desired amounts of sodium acetate buffer (pH=4.8) with a solid-to-liquid ratio of 1:20 (g/mL) and cellulase (35 FPU/g substrate). Then the mixture was incubated at 50 ^o^C in a rotary shaker with a rotational velocity of 150 rpm for 48 h. Next, the mixture was centrifuged, and the residue was washed thoroughly with sodium acetate buffer (pH=4.8) to remove the hydrolysed carbohydrates, and then freeze-dried. Finally, the dried residual solid was repeatedly subjected to ball-milling for 2 h and enzymatic hydrolysis again as the above-mentioned processes. After washing with acidic water (pH=2.0) and freeze-drying, a DEL sample was obtained.

**Note S2.** **Flocculation for kaolin suspension.**

The DES-based flocculant solution (1 mg/mL) and kaolin suspension (0.1 wt%, particle size = 5 μm) were freshly prepared before each flocculation test. The pH value of the kaolin suspension (100 mL) was adjusted with 0.1 M NaOH solution or HCl solution, and then a certain amount of flocculant was added to the kaolin suspension. The mixture was stirred rapidly at room temperature (400 rpm) for 1 min, then slowly (100 rpm) for 3 min. After standing for 30 min, the supernatant was collected and used to measure OD_600_ (optical density at 600 nm) using a UV-2450 spectrophotometer (Shimadzu, Japan) and zeta potential using Malvern Nano-Z Zetasizer, respectively. The removal rate of kaolin suspension by DES-based flocculant was calculated, as shown in Equation (1).

Removal rate (%) = (OD_0_-OD)/OD_0_ × 100% Equation 1

Where OD_0_ is the initial OD_600_ of kaolin suspension, and OD is the OD_600_ of supernatant after flocculation.

**Note S3. Flocculation and sterilisation for *E. coli*** **suspension.**

*E. coli* (CMCC 44102) was cultured in liquid Luria Broth LB medium (LLB, Difco; LB Broth, Miller) at 37 ^o^C for 24 h in 500 mL sterilised glass ﬂasks before ﬂocculation tests. LLB was prepared by dissolving 25 g of LB powder into 1 L of water, which was sterilised in an autoclave at 121 ^o^C for 15 min before use. The synthetic wastewater was prepared by harvesting the cultured bacteria by centrifugation at 3000 rpm for 5 min, followed by dilution and dispersal in deionised water to obtain a stable *E. coli* suspension.

A certain amount of flocculant solution (1 mg/mL) and *E. coli* suspension were added to the beaker. After that, a mechanical stirring device was used to stir it at 400 rpm for 1 min, 100 rpm for 3 min, and then stood for 30 min. The OD_600_ of *E. coli* suspension before and after flocculation was measured by UV-2450 spectrophotometer, which was expressed by (OD_0_) and (OD), respectively. The removal rate was calculated as shown in Equation (2).

Removal rate (%) = (OD_0_ − OD)/OD_0_ × 100% Equation 2

In addition, the zeta potential, three-dimensional excitation-emission matrix spectra of the supernatants were recorded for further analysis of the flocculation mechanism and sterilisation performance using Malvern Nano-Z Zetasizer and Hitachi F-7100 fluorescence spectrophotometer, respectively.

**Note S4.** **Adsorption test.**

The stock solutions of Pb^2+^, Zn^2+^ and Cu^2+^ were prepared by dissolving Pb(NO_3_)_2_, Zn(NO₃)₂ and CuSO₄ in ultrapure water, respectively, then diluted to the desired concentration. The adsorption isotherms were investigated by evaluating the adsorption capability of Pb^2+^ with 0.01 g adsorbent C-350 at various initial concentrations of Pb^2+^ (0-350 mg/L) at 25 ^o^C. The adsorption kinetics experiments were performed using 0.01 g C-350 at different time (0-720 min). The influence of initial pH on the adsorption capacity of C-350 was investigated in the pH range of 2-8. The impact of ionic strength on the removal of Pb^2+^ was also examined in 0, 0.005, 0.010, 0.015 and 0.020 mol/L KNO_3_. Then, the solid-liquid mixture was filtered through a 0.22 um nylon filter. The residual Pb^2+^ concentration in the supernatant was tested by a flame atomic absorption spectrophotometer (FAAS, A3F-13, PERSEE, China). Adsorption capacity (Q_e_) was calculated by the following equations (3).

Q_e_ = (C_0_ − C_t_) × V/m  Equation 3

Where C_0_ and C_t_ represent the initial and equilibrium concentrations of heavy metal ions in solution (mg/L); V is the solution volume (L) and m is the mass of adsorbent (g).

**Note S5.** **Photodegradation test.**

The performance of the photocatalyst was evaluated on photodegradation of Methyl orange (MO) using a 300-Watt Xenon lamp (ASASHI SEPECTRA, Japan) used as a simulated sunlight source (2.5 kW/m^2^), and extended to methylene blue (ME), naproxen (NPX), acyclovir (ACV) and bisphenol A (BPA). In photodegradation tests, 15 mg catalysts were added to 30 mL of simulated wastewater (10 mg/L). The adsorption-desorption equilibrium between catalysts and pollutants was established through the magnetic stirring of the suspension in the dark for 30 min. During the experiments, 1.5 mL suspension was collected with 5 mL syringe at predetermined intervals and filtered through a 0.45 μm PALL syringe filter. The concentrations of MO and ME were measured by UV-2450 spectrophotometer at 276 nm and 665 nm, respectively. Ultrahigh-performance liquid chromatography (UPLC, Primaide, HITACHI, Japan) was utilised to monitor the concentrations of NPX, ACV and BPA with a UV detector. The retention time was 6.5 min for NPX after chromatographic separation on column (Agilent ZORBAX SB-C18, 3.5-micro, 4.6 × 75 mm, USA) at 230 nm and a constant 30 ^o^C column temperature. The mobile phase (2% acetic acid in water: methanol: acetonitrile = 55: 40: 5) was delivered at 1 mL/min and the injection sample volume was 20 μL. The retention time was 10 min for ACV after chromatographic separation on column (Agilent ZORBAX SB-C18, 3.5-micro, 4.6 × 75 mm, USA) at 254 nm and a constant 30 ^o^C column temperature. The mobile phase (methanol: water = 5: 95) was delivered at 1 mL/min and the injection sample volume was 20 μL. The retention time was 5 min for BPA after chromatographic separation on column (Agilent ZORBAX SB-C18, 3.5-micro, 4.6 × 75 mm, USA) at 224 nm and a constant 30 ^o^C column temperature. The mobile phase (methanol: acetonitrile = 50: 50) was delivered at 1 mL/min and the injection sample volume was 20 μL. Degradation efficiency (DE) was calculated by the following equations (4):

DE = (C_0_-C_t_) ˟ V/(M ˟ m ˟ t) Equation 4

Where C_0_ and C_t_ represent the initial and t time concentrations of pollutant in solution (mg/L); V is the solution volume (L), M is the molecular weight of the pollutant, m is the mass of adsorbent (g), and t is the sampling time (h).

In addition, the effect of catalyst dosage on photodegradation was evaluated at dosages of 0.05, 0.25, 0.5, and 1 mg/mL. Moreover, to understand which active species played a primary role in the MO degradation process with CN-1, various shielding tests for photogenerated holes and free radicals were performed. The ammonium oxalate ((NH_4_)_2_C_2_O_4_) was used as a quencher for holes (h^+^). The N_2_ atmosphere was used to shield superoxide radicals (•O− 2), while butylated hydroxytoluene (BHT) and isopropanol (IPA) were quenchers for alkyl radicals (•R) and hydroxyl radicals (•OH), respectively.

**Note S6. The chemical compositions of the cellulose-rich substrates.**

The chemical compositions (wt%) of the cellulose-rich substrates were determined according to the NREL standard analytical method (NREL/TP-510-42618)^1^.

**Note S7. The equations for pretreatment performance analysis.**

Solid yield (%) = Solid residue (g) /Raw material (g) × 100% Equation 5

Delignification ratio (%) = (1- Lignin in solid residue after pretreatment (g) /Initial amount of lignin in the raw material (g)) × 100% Equation 6

**Note S8. Characterisation** **methods.**

**FT-IR Spectroscopy**: FT-IR spectra of samples were obtained using a Thermo Scientiﬁc Nicolet iN10 FT-IR microscope (Thermo Nicolet Corp., Madison, WI, USA) equipped with a liquid nitrogen-cooled MCT detector. The dried lignin samples were ground and pelletised using BaF2, and their spectra were recorded in the range from 4000 to 700 cm^−1^ at 4 cm^−1^ resolution and 128 scans per sample.

**Zeta potential**: First, a 10 mg sample was added to 10 mL of deionised water. Then the solution was adjusted to the desired pH using 0.1 M HCl or NaOH solution, and the solution was taken to measure the zeta potential by Malvern Nano Z (Malvern, Malvern Instruments Ltd., UK).

**NMR spectra**: The ^1^NMR spectra were recorded on a Bruker AVIII 400 MHz (Bruker, Germany) spectrometer at 25 ^o^C, and a 20 mg sample was dissolved in 0.5 mL of DMSO-*d_6_* (99.8% D). The peak detected using 2.49 ppm of DMSO-*d_6_* was used as the internal standard. In terms of 2D-HSQC spectra, 50 mg of lignin was dissolved in 0.5 mL of DMSO-*d_6_*, and the Bruker standard pulse program hsqcetgp was used for 2D-HSQC experiments. The spectral widths were 5000 and 20000 Hz for the ^1^H- and ^13^C-dimensions, respectively. The quantitative ^31^P NMR spectra were adopted to monitor the content of aliphatic and phenolic hydroxyl groups in the lignin^2,3^. The analysis was performed on lignin samples phosphylated with 2-chloro-4,4,5,5-tetramethyl-1,3,2-dioxaphospholane, using cyclohexanol as an internal standard. A dried lignin sample (20 mg) was dissolved in 500 μL of an anhydrous pyridine/CDCl_3_ mixture (1.6:1, v/v). Here, 100 μL of chromium(III) acetylacetonate and 100 μL of cyclohexanol served as the relaxation reagent and internal standard, respectively.

**Specific surface area:** The specific surface area of samples was recorded by analysing the nitrogen adsorption using a surface area analyser (Kubo-X1000, China) after 8 h of degassing at 120 ^o^C.

**Gel permeation chromatography (GPC)**: The weight-average (*M*_w_) and number-average (*M*_n_) molecular weights of the lignin samples were determined by gel permeation chromatography (GPC) (Agilent 1200, Agilent Technologies, USA) with an ultraviolet detector (UV) at 240 nm. The column used was a PL-gel 10 mm mixed-B 7.5 mm i.d. column, which was calibrated with PL polystyrene standards according to a previous report.^4^ For DES-based flocculants, 4 mg DBF was dissolved in 2 mL of 5% acetic acid solution. The solution was stirred at 300 rpm for 6 h. After that, the solution was ﬁltered using 0.2 μm nylon ﬁlter. The solution, after ﬁltration, was injected into a gel permeation chromatography system, Malvern GPCmax VE2001 Module and Viscotek TDA305 with multidetectors (UV, RI, viscometer, low angle and rightangle laser detectors) for assessing the molecular weight. A 0.60 mL/min ﬂow rate of 5% acetic acid solution was used with columns PAC103 and PAC101 in this system. The column temperature was set at 35 ^o^C, and pullulan, 47 300 g/mol, was used as standard.

**The surface morphologies**: The morphology and elemental mapping were recorded on a JEOL JSM- 7800F Field Emission Scanning Electron microscope coupled with a Thermo Scientific Ultra-dry EDS detector. The morphologies were observed by a high-resolution transmission electron microscope (HRTEM, JEMF200, JEOL, Japan), and the particle size statistics were performed using Nano Measure 1.2.5 software (Fudan University, China). The HRTEM images were processed by Gatan Digital Micrograph software to obtain fast Fourier transform images.

**X-ray photoelectron spectroscopy (XPS)**: X-ray photoelectron spectroscopy (ESCALAB 250Xi, USA) was used to measure the carbon (C), oxygen (O) and nitrogen (N) content and functional group composition on the surface of the CNs.

**Photoluminescence (PL)**: The fluorescence spectrophotometer (Hitachi F-7100, Japan) was used to measure the photoluminescence with 350 nm excitation wavelength.

**UV-vis diﬀuse reflectance spectra (DRS)**: The UV-vis spectrophotometer (Shimadzu UV-3600, Japan) was used to measure the UV-vis diﬀuse reflectance spectra.

**Electron paramagnetic resonance (EPR)**: The electron paramagnetic resonance spectrometer (Bruker A300-10/12, Germany) was used to measure electron paramagnetic resonance spectra at 77 K.

**Note S9. Photoelectrochemical test.**

Photoelectrochemical testing was completed on an electrochemical workstation (Austin CHI-760E, USA) using a xenon lamp-assisted three-electrode electrochemical testing system. A platinum wire was used as the counter electrode, the reference electrode was a saturated calomel electrode, and 0.1 mol/L sodium sulfate solution was used as the electrolyte. The photocatalytic material powder was coated on indium tin oxide conductive glass (ITO) as a working electrode. The specific coating method of the working electrode was to add 8 mg of catalyst powder to 0.6 mL Nafion117 ethanol dilution, and after ultrasonic treatment for 20 minutes, the suspension was evenly dropped onto the ITO conductive glass with a clean surface using a pipette. After the liquid has completely evaporated, place it in a blast drying and heat it at 120 °C for 2 h. The light source used in the test was a 300W high-pressure xenon lamp with a 420 nm cutoff filter.

**2. Results**

**Note S10.** **Evaluation of DES pretreatment** **process.**

XR is a solid byproduct derived from corn cobs through mild acidic hydrothermal treatment for producing xylo-oligosaccharides, which is a typical industrial waste biomass. The current xylose industry only converts hemicelluloses into xylo-oligosaccharides, leaving a large amount of XR rich in cellulose and lignin, which has not been well developed and utilised due to the recalcitrant and intricate structures of XR^5^. In China alone, the annual XR production reaches as high as 40 million tons, and the demand continues to grow rapidly, while a significant portion being low-value were burned for heat, resulting in environmental pollution and wastage of lignocellulosic resources^6^. Therefore, using XR for water remediation will not only enhance the valorisation of industrial renewable waste but also contribute to the development of environmentally sustainable wastewater treatment methods.

In this study, an easy-to-operate DES pretreatment strategy was employed to mitigate the biomass recalcitrance to efficiently deconstruct XR, and endow the main components (cellulose and lignin) with functionality, facilitating the subsequent preparation of wastewater remediation materials, as shown in **Fig. 2a**. DES was composed of mixing acrylic acid (AA), methyl acryloyloxyethyl trimethylammonium chloride (MTAC) and aluminium chloride at the molar ratio of 1:1:0.05. Considering the significant influence of pretreatment temperature on the deconstruction efficiency and the functionalisation, this study conducted functionalisation pretreatments of the XR at various temperatures (70, 90, 110, and 130 ^o^C). As shown in **Table S1**, with the increase in pretreatment temperature, the solid yield obtained after DES pretreatment decreased from 63.85 to 53.71%, primarily due to the removal of lignin (delignification up to 88.37%), signifying that an appropriately elevated temperature facilitated the mitigate the biomass recalcitrance and further lignin removal **(Fig. 2b)**. However, as the pretreatment temperature continued to rise to 130 ^o^C, the delignification rate slightly decreased to 82.62% due to the lignin condensation at high temperature. Therefore, evaluating the pretreatment effectiveness based on the delignification rate, the optimal pretreatment temperature was 110 ^o^C. In addition, cellulose-enriched residue was analysed using FT-IR technology to demonstrate that cellulose was functionalised alongside the delignification process. As shown in **Fig. 2c**, as compared with raw XR, the lignin-specific signal peaks (1599, 1513, 1263 cm^-1^) were significantly attenuated or even disappeared in the FT-IR spectrum of R-110, confirming once again the high delignification rate. Notably, the C=O stretching vibration peak at the carboxyl group (1717 cm^-1^) was significantly enhanced, indicating the introduction of carboxyl groups onto the cellulose^7^. The enhanced electrostatic repulsion and hydrophilicity caused by surface carboxyl groups would facilitate the preparation of LCNF from cellulose-enriched residue, which was an excellent in-situ carbon doping source and biological template for the preparation of carbon nitride-based photocatalysts.

To unveil the lignin functionalisation during pretreatment, a comprehensive structural elucidation was conducted through FT-IR, ^1^H NMR, ^31^P NMR, 2D-HSQC NMR and GPC techniques. Besides, double enzymatic lignin (DEL), as the lignin model, was used for comparison with the lignin components obtained by DES pretreatment, which helped to explore the functionalisation of lignin. In the side-chain region of 2D-HSQC spectra **(Fig. S1a)**, a distinctive pattern emerged wherein only signals attributable to aryl ether linkages (A, β-*O*-4) and acylated aryl ether linkages (A', β-*O*-4) were manifest^6^. This was because the linkages of lignin were damaged to a certain extent in the acidic-high temperature environment during the xylo-oligosaccharides production. It was found that the signal strengths of aryl ether bond and acylated aryl ether bond decreased significantly after DES pretreatment, and became weaker with the increase of pretreatment temperature. This phenomenon emphasised that DES pretreatment could degrade ether linkages in lignin, thereby promoting the removal of lignin from XR. As elucidated in **Table S2**, a corresponding decrease in molecular weight with increasing pretreatment temperature was observed. Moreover, a new β-β resinols (B) signal appeared in the lignin components after pretreatment^8^, and its intensity was positively correlated with the pretreatment temperature. This emergence was ascribed to the fact that lignin would partially condense to form carbon-carbon bonds at high pretreatment temperatures^9^, which echoed that the excessive temperature would reduce the delignification. In regard to the aromatic region, minimal deviations were detected between the lignin components and DEL, attesting that the basic structural units of lignin were not destroyed during the pretreatment process **(Fig. S1b)**. However, the signal intensities of almost all structural units of lignin after pretreatment were weakened, and they were weaker with the increase in pretreatment temperature. This phenomenon might be caused by the introduction of part of DES into lignin, which made it less soluble in DMSO.

As depicted in **Fig. S2** and **Table S2**, the ^31^P NMR results afforded a succinct revelation: the lignin components exhibited a significant decrease in the alcoholic hydroxyl content, while the carboxyl content increased. This indicated that DES pretreatment modified lignin by carboxylation during delignification, and the degree of carboxylation increased with the increase of pretreatment temperature. This conclusion was also supported by the gradual intensification of the stretching vibration peak attributed to C=O at 1710 cm^-1^ in the FT-IR of lignin components^10^ **(Fig. S3a)**. In addition, a new absorption peak appeared at 1460 cm^-1^ caused by C-H bending vibrations on the quaternary ammonium salt^11^, which preliminarily proved that lignin introduced quaternary ammonium salt groups. Moreover, in the ^1^H NMR spectra **(Fig. S3b)**, in addition to the aromatic proton signal peaks (6.21-7.60 ppm) and methoxyl signal peaks (3.5-3.97 ppm) from lignin, a strong new peak appeared at 3.05-3.22 ppm, which was attributed to the quaternary ammonium salt proton peak^12^.

The results showed that a large number of carboxyl and quaternary ammonium salt groups were introduced into the lignin skeleton during the delignification process from the analysis results of FT-IR and ^1^H NMR. The carboxyl groups of L-110 lignin were as high as 1.06 mmol/g and were 7 times the carboxyl group content of DEL, which was due to the introduction of DES into lignin. It is well known that abundant carboxyl groups play a vital role in promoting the adsorption capacity of lignin for cationic pollutants such as heavy metal ions. In addition, quaternary ammonium salt groups on lignin would produce a mass of gas during low-temperature calcination, which provided a guarantee for the green preparation of porous biochar. In general, the DES pretreatment process could not only effectively remove lignin from XR, but also endowed lignin with carboxyl functional groups and quaternary ammonium salt groups. The lignin functionalisation provides an advanced structural matrix that is particularly beneficial for the subsequent deployment of lignin-derived biochar to adsorb heavy metals.

Although DES provide a cost advantage over ionic liquids, the industrial application in effluent faces hurdles. This study aims to reduce DES production costs by exploring the optimal conditions for DES recycling, emphasising its importance in the industrial context of wastewater treatment. As shown in **Fig. 2e**, the ability of DES to deconstruct XR sustained a relatively high delignification rate in the initial four cycles, while the delignification rate and cycle efficiency sharply decreased to 75.32% and 85.23% in the fifth cycle. This attenuation was attributed to the challenge of isolating the lignin fragments from DES and the inevitable loss of acrylic acid during the DES recycling process, disrupting the hydrogen-bonding constitution of the DES system. In the industrial sector, the use and disposal of solvents often lead to waste streams, which in turn increases the costs associated with solvent disposal. Nevertheless, DES amassed a substantial concentration of lignin fragments after undergoing multiple cycles of usage, which will provide a priori condition for the copolymerisation of DES mixtures to produce efficient flocculants at ambient temperature and pressure. This pioneering method has the prospect of diminishing the costs associated with industrial waste solvent disposal whilst simultaneously providing an opportunity for generating new materials. As such, this approach augments resource efficiency and embodies the ethos of waste reduction.

**Note S11. Characterisation analysis for DES-based flocculant.**

As depicted in **Fig. S4a**, the FT-IR spectrum of DBF-25 revealed distinctive peaks attributing to aromatic rings at 1599 and 1232 cm^-1^, a stretching vibration peak corresponding to C=O on carboxyl at 1720 cm^-1^, and a bending vibration peak of C-H on quaternary ammonium salts at 1473 cm^-1 13,14^. Moreover, compared to DES, the ^1^H NMR result of DBF exhibited signals from methoxy groups (3.85-3.97 ppm) and aromatic protons (5.75-6.57 ppm) originating from lignin fragments^12,15^, as shown in **Fig. S4b**. These phenomena indicated that the degradation products of lignin could not only establish a self-catalytic system with Al^3+^, but also participate in the copolymerisation reactions of AA and MTAC. It is well-known that charge density and molecular weight play crucial roles in the flocculation process^16^. **Fig. S5a** demonstrated that DBL-25 had a high positive zeta potential, which could attract oppositely charged colloids through charge attraction, thereby accelerating colloid precipitation. In addition, **Fig. S5c** showed that DBL-25 had a high molecular weight of 139,940 g/mol, which was conducive to enhancing the bridging effect during the flocculation process.

To delve into the internal factors of the flocculant, a series of DES-based flocculants (DBLs) were prepared at different temperatures. As shown in **Fig. S4a**, DBLs prepared at different temperatures displayed nearly identical FT-IR spectra. Comparing the charge densities and molecular weights of different samples revealed that those prepared under lower reaction temperature conditions had higher positive zeta potential (**Fig. S5a**) and molecular weight (**Fig. S5c**). The positive charge groups in DBL came from the quaternary ammonium salt on MTAC, while the carboxyl groups on AA provided the negative charge groups. It could be hypothesised that AA primarily participated in the copolymerisation reaction at higher temperatures, while lower temperatures favoured the copolymerisation of MTAC.

**Note S12. Flocculation evaluation for kaolin suspension.**

In the initial stage, the removal rate of kaolin and the zeta potential of the supernatant rapidly increased with the increasing dosage of DBL-25 (**Fig. 3c)**. When the dosage of DBL-25 reached 4 mg/L, the optimal dosage was reached, and the removal rate of kaolin exceeded 99%. Notably, at this point, the zeta potential of the supernatant was -6.3, which contradicted the classical charge neutralisation theory^17^. This suggested the presence of a charge patching effect in the flocculation process^18^, likely due to the carboxyl groups in DBL-25. Upon further dosage increase beyond the optimum, a phenomenon of anti-flocculation emerged, where the zeta potential continued to increase, and the removal rate decreased. This was because kaolin particles adsorbed excessive flocculants with a positive charge, increasing electrostatic repulsion among particles and reducing flocculation efficiency. Therefore, it could be inferred that the flocculation mechanism involved in the process of DBL-25 flocculating kaolin suspension included charge attraction (charge neutralisation and charge patching) and bridging effect.

In addition, external factors such as pH and coexisting ions in actual wastewater would significantly affect flocculation efficiency^18,19^. As depicted in **Fig. S6a**, DBF-25 demonstrated superior flocculation performance on kaolin suspension under acidic conditions. This was attributed to the abundance of hydrogen ions in acidic conditions, leading to protonation and thereby enhancing the flocculation effect^20^. When the wastewater pH was 9, DBF-25 still exhibited respectable flocculation ability. Under conditions of strong alkalinity (pH=11), the flocculation effect of DBF-25 was attenuated due to the process of deprotonation, but the removal efficiency still exceeded 93%. **Fig. S6b** demonstrated that coexisting cations (Ca^2+^ and Mg^2+^) did not affect the flocculation efficiency of DBF-25. Concerning coexisting anions, DBF-25 exhibited excellent resistance to NO_3_^-^ (**Fig. S6c**). However, the existence of SO_4_^2-^ weakened the flocculation effect of DBF-25 to some extent, resulting from competition adsorption between SO_4_^2-^ and kaolin particles. Overall, DBF-25 demonstrated robust resistance to external factors like wastewater pH and coexisting ions, making it suitable for a wide range of scenarios.

**Note S13. Flocculation and sterilisation evaluation for E. coli suspension.**

As shown in **Fig. 3e**, when the dosage of DBF-25 was less than 30 mg/L, the zeta potential and removal rate of the supernatant increased rapidly as the dosage increased. At the optimal dosage of 30 mg/L, DBF-25 achieved a removal rate of 99.5% for the *E. coli* suspension. Interestingly, as the dosage continued to increase, the supernatant's removal rate and zeta potential remained stable without showing anti-flocculation, which did not align with the conventional flocculation process. This suggested that, in addition to flocculation, other mechanisms were at play in this process. Through the three-dimensional fluorescence spectrum (3D EEM) of the supernatant shown in **Fig. 3f**, significantly enhanced signals belonging to soluble products (200-240/280-380 nm) and internal aromatic proteins (250-300/300-400 nm) of *E. coli* were detected in the supernatant^15^. Generally, these signals could be detected in small amounts due to the metabolism of *E. coli*^21^. However, these signals were detected to be significantly enhanced when the dosage of DBF-25 exceeded 30 mg/L, indicating that the *E. coli* cell structure was disrupted. Moreover, the intensities of these signals increased with increasing dosages of DBF-25. These phenomena illustrated that when DBF-25 purified *E. coli* suspension, it was mainly used for flocculation when the dosage was small, but when there was enough DBF-25, the sterilisation effect could be clearly activated.

**Note S14. Characterisation analysis for lignin-based biochar.**

As depicted in **Fig. 4a**, the FT-IR spectrum of C-350 still exhibited a stretching vibration attributable to the C=O bond of carboxyl groups at 1717 cm⁻¹^7^, suggesting that carboxyl groups on the lignin were retained during the low-temperature annealing process. Additionally, the C-H bending vibrations on the quaternary ammonium salt at 1460 cm⁻¹ decreased sharply^11^, indicating that the quaternary ammonium group was seriously pyrolysed. The thermal cracking of quaternary ammonium salts would produce a large amount of gas, which helped to create pores during the annealing process and increase the specific surface of the prepared biochar. BET results in **Fig. S7a** confirmed this conclusion, and the specific surface area sharply increased from 1.03 m^2^/g of L-110 to 40.33 m^2^/g of C-350 after low-temperature annealing. By comparing the surface morphology of the samples before and after annealing (**Figs. 4b** and **4c**), the surface of the precursor L-110 was smooth, while the C-350 showed a porous structure. The rich carboxyl groups and porous structure would ensure the efficient adsorption of heavy metal ions by C-350.

The annealing temperature had a decisive impact on the functional group composition, specific surface area and yield of biochar^22,23^. In order to explore the influence of internal factors on the adsorption capacity of biochar, a series of lignin-based biochars were prepared in the range of 250-700 ^o^C. Apparently, the yield of biochar decreased with increasing annealing temperature due to the cleavage of linkages and functional groups (**Fig. S7b**). As shown in **Fig. S7c**, as the annealing temperature increased, the carboxyl signal intensity (1717 cm^-1^) on the FT-IR of biochar gradually weakened, and almost no signal peaks belonging to carboxyl groups were observed until the annealing temperature exceeded 500 ^o^C. Moreover, **Fig. S7a** showed that the specific surface area of biochar increased rapidly with increasing annealing temperature in the range of 250–350 ^o^C. When the annealing temperature was higher than 350 ^o^C, due to a serious lack of gas production, the specific surface area of biochar decreased, even lower than the specific surface area of L-110. Combining the results of carboxyl content and specific surface area, it could be inferred that C-350 had the highest adsorption capacity.

**Note S15. Adsorption evaluation for heavy metals.**

As shown in **Fig. 4e**, the adsorption capacity of C-350 for Pb^2+^ increased with higher initial Pb^2+^ concentrations. Data fitting using Langmuir and Freundlich isotherm models revealed that the adsorption of Pb^2+^ by C-350 was more in line with the Langmuir isotherm model (**Table S3**), which indicated that the adsorption process belonged to monolayer adsorption^24^. The maximum adsorption capacity of C-350 for Pb^2+^ was as high as 143.21 mg/g calculated by the Langmuir isotherm model, which was superior to the majority of biochars reported in the literatures^25,26^. **Fig. S8** visually presented the uniform adsorption of Pb^2+^ onto the surface of C-350 through SEM images and corresponding elemental mapping analyses. Moreover, the effect of adsorption time on the adsorption capacity of C-350 is depicted in **Fig. S9a**. In the initial stage of the adsorption process, the adsorption capacity of C-350 increased rapidly. However, the growth rate of adsorption capacity markedly declined with prolonged time. Based on this, the kinetic model of C-350 adsorption of Pb^2+^ was fitted, and the results showed that the correlation coefficients (R^2^) of the pseudo-first-order model and pseudo-second-order model were 0.9479 and 0.9857, respectively, both showing outstanding fitting results. This indicated that the adsorption process involved both physisorption and chemisorption^27^.

The influence of external factors, such as wastewater pH and ionic strength, on the adsorption capacity of C-350 was also explored. As shown in **Fig. S9b**, the adsorption capacity of C-350 was significantly reduced in a strongly acidic environment. This was because the protonation would increase the electrostatic repulsion between C-350 and Pb^2+^, reducing the adsorption capacity of C-350^28^. As the pH value increased, the adsorption capacity of C-350 increased due to the ionisation of carboxyl groups^29^. When the pH value reached 8, the presence of a large number of hydroxide ions would promote the precipitation of Pb^2+^. The effect of ionic strength on the adsorption capacity of C-350 is shown in **Fig. S9c**. When the wastewater pH was 2, the adsorption capacity of C-350 decreased with the increase of ionic strength, while at pH 5.78, the ionic intensity had a negligible impact on C-350 adsorption capacity. These results indicated that under strong acid conditions, the adsorption of Pb^2+^ by C-350 was carried out by ion exchange at permanently negative potential sites^30^. The presence of cations would compete with Pb^2+^ for adsorption, thereby reducing the adsorption capacity of C-350. However, when the wastewater pH was 5.78, various electron-donating groups of C-350 gradually became the main adsorption sites, and Pb^2+^ were adsorbed on the surface of C-350 through chelation^31^, thereby greatly weakening the influence of ionic strength on adsorption.

Apart from Pb^2+^ wastewater, this study also deeply explored the adsorption capacity of C-350 on Zn^2+^ wastewater, Cu^2+^ wastewater and Pb^2+^-Zn^2+^-Cu^2+^ mixed wastewater. As shown in **Fig. S10a** and **Table S3**, the maximum adsorption capacities of Zn^2+^ and Cu^2+^ by C-350 were 110.64 mg/L and 64.84 mg/L, respectively, both of which were consistent with the Langmuir isotherm model. **Fig. S10b** showed that C-350 could effectively remove three metal ions for the Pb^2+^-Zn^2+^-Cu^2+^ mixed wastewater at the same time, which held great promise for the purification of complex aquatic environments in practical wastewater treatment scenarios.

**Note S16. Characterisation analysis for photocatalyst.**

As shown in **Fig. S11a**, the high-resolution C 1s spectrum of the pristine CN could be deconvoluted into four peaks at 284.8, 286.63, 288.02, and 293.54 eV, attributed to the C-C/C=C adventitious carbon species, C-N-C sp^2^ hybridisation, N=C-N sp^3^ hybridisation, and π-π* excitation between graphite layers, respectively.^32,33^ The C 1s high-resolution spectra of CN-1 and CN-CNF showed new peaks around 288.4 eV, attributed to C-O, resulting from slight oxidation by water and carboxyl groups on cellulose during the thermal condensation process. A comparison revealed that the signals of C-C and C-N-C slightly increased in CN-1 and CN-CNF compared to pristine CN, which was attributable to the successful incorporation of carbon atoms from LCNF and CNF. **Fig. S11b** showed that the three samples had almost the same N 1s XPS spectrum. As shown in **Figs. S12a** and S**12b**, the FE-TEM images of pristine CN revealed a two-dimensional sheet-like structure, while CN-1 exhibited a nanotube structure, indicating that LCNF played a role as a bio-template to some extent. Moreover, BET results indicated that the addition of LCNF and CNF increased the specific surface area (**Fig. S12c**). The nanotube structure and higher surface area were advantageous for light absorption and scattering, improving the adsorption of organic pollutants and inducing efficient charge separation^34^.

The optical properties of CN, CN-1, and CN-CNF were investigated to understand further the impact of LCNF and CNF incorporation on the photocatalytic performance of g-C_3_N_4_. As shown in **Fig. 5c**, the ultraviolet-visible diffuse reflectance spectra (DRS) revealed absorption edges at 460, 486, and 475 nm for CN, CN-1, and CN-CNF, respectively, indicating carbon doping enhanced the light absorption ability. Moreover, CN-1 exhibited stronger light absorption than CN-CNF, primarily attributed to the highly condensed lignin with excellent light-absorbing ability contained in LCNF. According to the Kubelka-Munk (KM) formula^35^, the bandgaps of CN, CN-1, and CN-CNF were calculated to be 2.69, 2.55 and 2.61 eV, respectively (**Fig. S13a**). **Fig. 5a** showed that the valence band (VB) energy levels of all three photocatalysts were higher than the standard redox potential of (OH−/•OH), while the conduction band (CB) positions were more negative than the reduction potential of (O_2_/•O− 2). The generation of •OH and •O− 2 would be beneficial for the degradation of pollutants in the water. Furthermore, CN-1 with a narrower bandgap, was expected to exhibit superior wastewater purification capability^36^. The electron structures of the three samples were compared by electron paramagnetic resonance (EPR) spectroscopy (**Fig. 5d** and **Fig. S14a**), which showed a single Lorentz line with a g value of 2.0039. This signal could be attributed to the π-conjugated aromatic delocalised structures of the photocatalysts^36^. CN-1 had a stronger EPR signal intensity, indicating higher defect density and a stronger π-conjugated system originating from more charged pairs, facilitating the migration of photogenerated charge carriers. Photoluminescence (PL) spectroscopy can reflect the capture, migration, and recombination of electron-hole pairs in semiconductors^37^. As shown in **Fig. S14b**, CN-1 exhibited the weakest peak in PL, indicating that the introduction of LCNF could alleviate the rapid recombination of electron-hole pairs, thereby making it easier for charge carriers to migrate to the photocatalyst surface. Additionally, as shown in **Fig. 5e**, CN-1 exhibited a higher photocurrent response than the other samples. Moreover, CN-1 had the smallest electrochemical impedance radius, indicating lower interface electron transfer resistance (**Fig. 5f**). These photoelectrochemical results again indicated that the introduction of LCNF contributed to the separation and transfer of photogenerated electron-hole pairs, and CN-1 showed stronger photocatalytic performance.

In addition, the effect of photocatalyst internal factors, namely the amount of LCNF added, on the catalyst structure and performance was also systematically investigated. As shown in **Fig. S15**, with the increase of LCNF addition, the bandgap of the photocatalyst gradually decreased. **Fig. S17** showed that when the mass of LCNF suspension was greater than or equal to 2.5 times that of urea, the resulting photocatalysts could not induce the generation of •OH, and CN-50 could not even induce the generation of •O− 2. **Figs. S18a and S18b** respectively showed that CN-1 exhibited a stronger photocurrent response and a smaller electrochemical impedance compared to the other samples.

**Note S17. Photodegradation evaluation for organic pollutants.**

As shown in **Fig. 5g**, CN-1 demonstrated the strongest photodegradation performance and was able to degrade almost all MO within 60 min. A comparison revealed that the degradation efficiency of CN-1 was 3 times higher than that of pristine CN (**Fig. S19**). The effect of the added amount of LCNF on the catalyst structure and photodegradation performance was also investigated.

As shown in **Fig. S20**, when the mass of LCNF suspension was greater than or equal to 2.5 times that of urea, the resulting photocatalysts exhibited worse photodegradation effects as the amount of LCNF introduced increased. Therefore, CN-1 was considered the optimal solution. Significantly, as compared to previous literature reports^33,36,38^, the ability to maintain the superior photodegradation with a low dosage of external carbon doping source in this study could be attributed to the excellent hydrophilicity, abundant carboxyl groups, and high surface area of LCNF. These properties allow for a thorough binding with urea in the aqueous solution, achieving effective doping without the need for excessive LCNF additions. **Fig. S21** exhibited that as the dosage of CN-1 increased, the degradation rate of MO accelerated. However, when the dosage of CN-1 exceeded 1 mg/mL, further increasing the CN-1 dosage had little effect on MO degradation because excessive photocatalyst affected light absorption. In addition, the photodegradation tests of Methylene blue, Naproxen, Bisphenol A and Acyclovir simulated wastewaters were carried out when the dosage of CN-1 was 0.5 mg/mL. The results in **Fig. S22** showed that CN-1 exhibited distinguished degradation effects on these four pollutants, demonstrating the outstanding universality of CN-1.

The photodegradation mechanism of MO by CN-1 was investigated. As mentioned above, CN-1 could theoretically guide the generation of •O− 2 and •OH, and EPR detection results confirmed this conclusion (**Fig. S23**). Moreover, various shielding tests for photogenerated holes and free radicals were performed. As shown in **Fig. 5h** and **Fig. S24**, the photodegradation ability of CN-1 to MO decreased severely under N_2_ atmosphere, followed by the addition of isopropanol (IPA), a scavenger for •OH, indicating that •O− 2 and •OH played critical roles in MO degradation. Additionally, as compared with the blank sample without a scavenger, it was found that alkyl radicals (•R) also played a certain role in the photodegradation of MO. Furthermore, the holes on the VB, which had a high oxidation potential, could directly participate in MO degradation^39^.


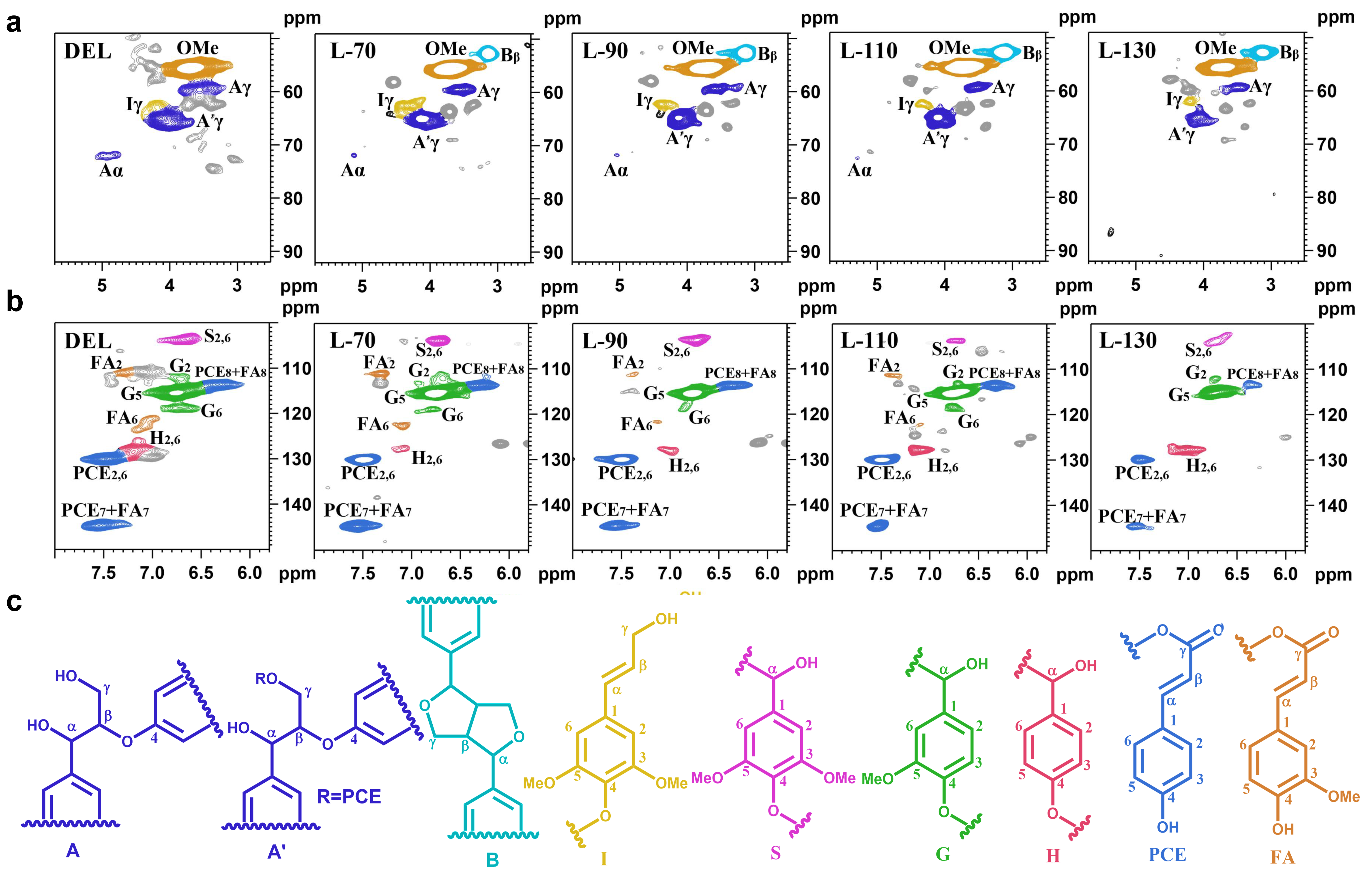


**Fig. S1.** 2D-HSQC spectra of the lignin samples. (a) Side-chain regions in 2D-HSQC spectra of the lignin samples. (b) Aromatic regions in 2D-HSQC spectra of the lignin samples. (c) The identified main structures of the lignin.


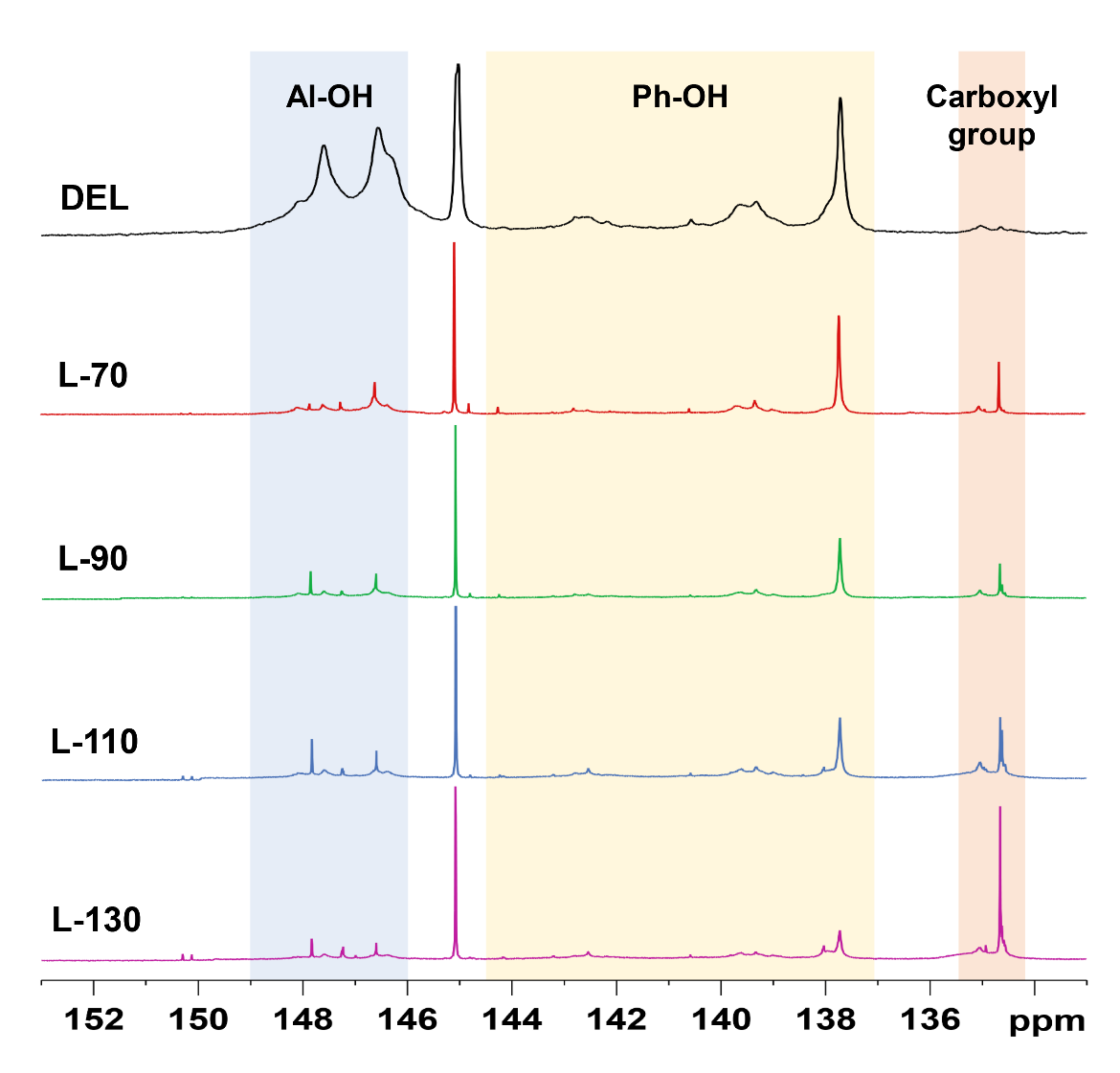


**Fig. S2.** ^31^P NMR spectra of the lignin samples.


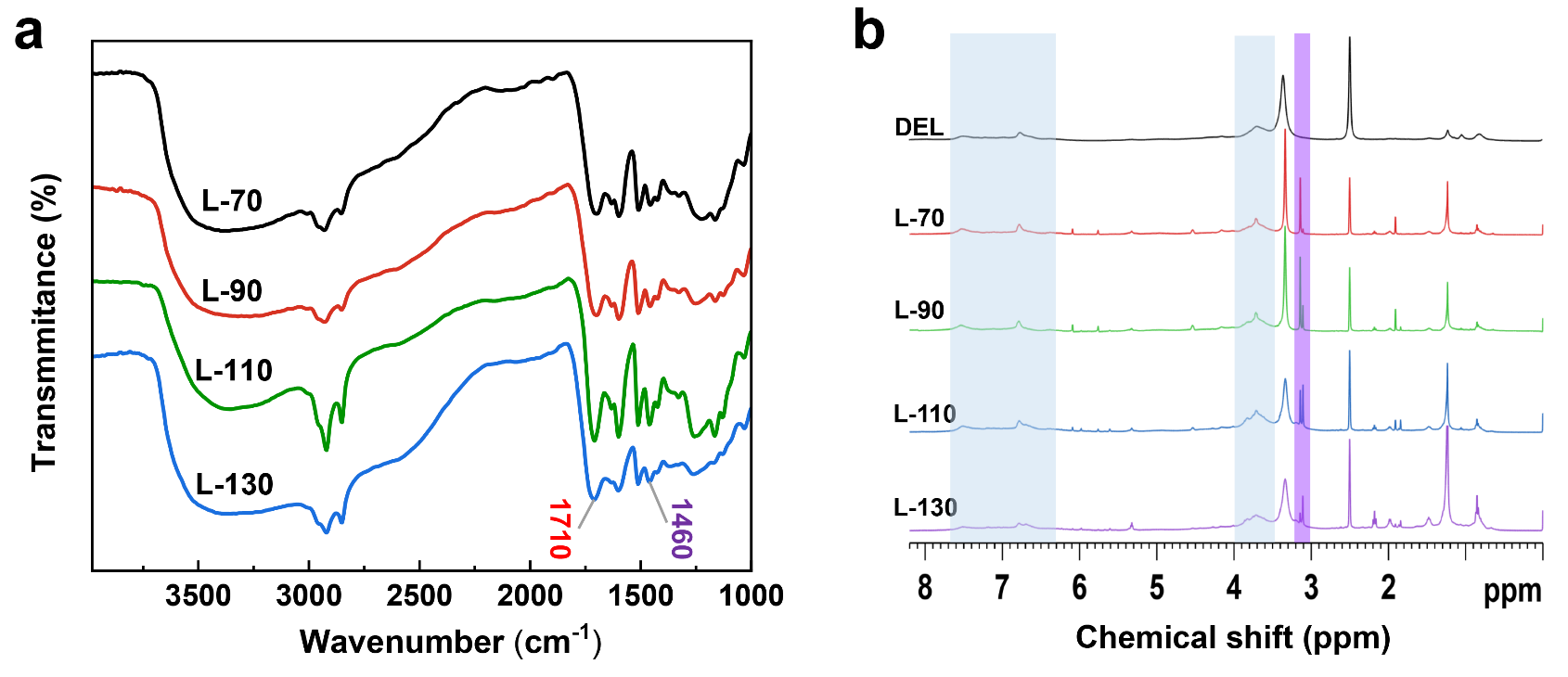


**Fig. S3.** FT-IR and ^1^H NMR spectra of lignin samples. (a) FT-IR and (b) ^1^H NMR spectra of lignin samples.


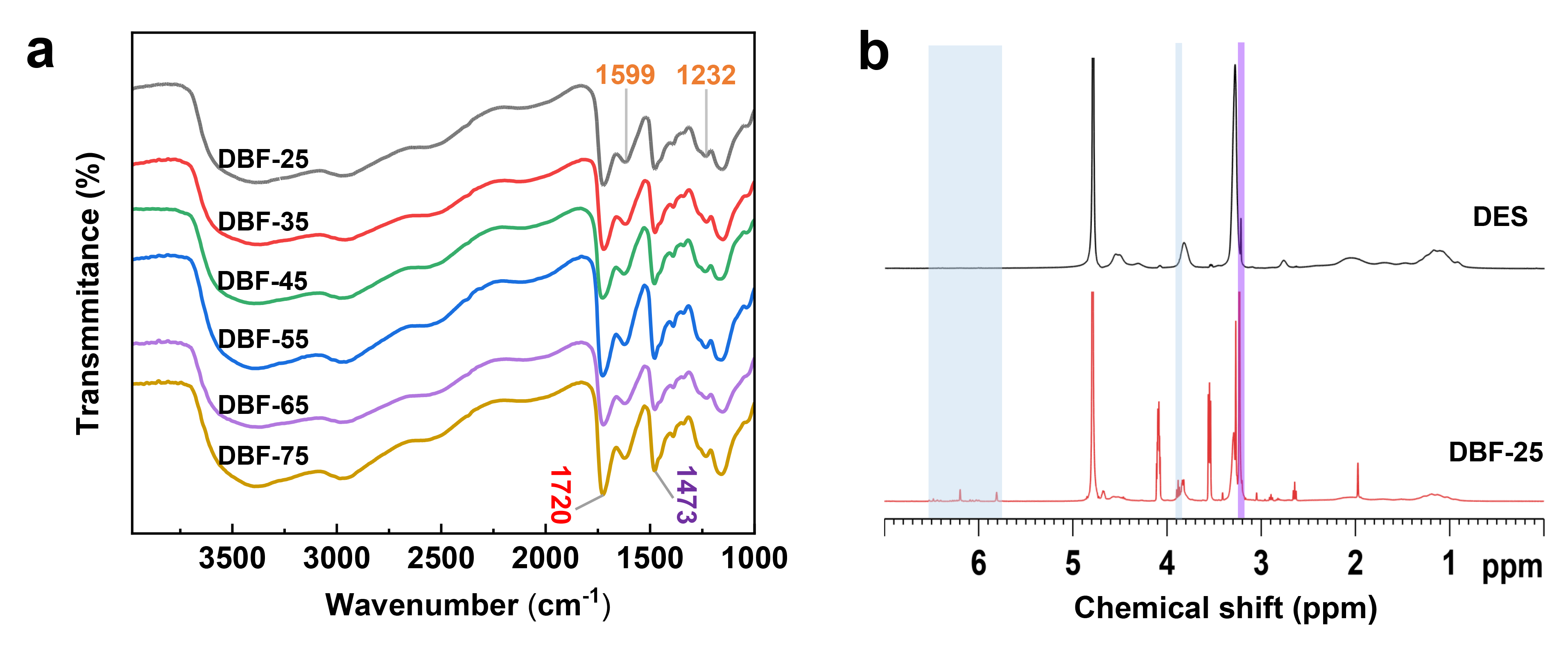


**Fig. S4.** FT-IR and ^1^H NMR spectra of flocculants. (a) FT-IR spectra of various DBF samples. (b) ^1^H NMR spectra of DES and DBF-25.


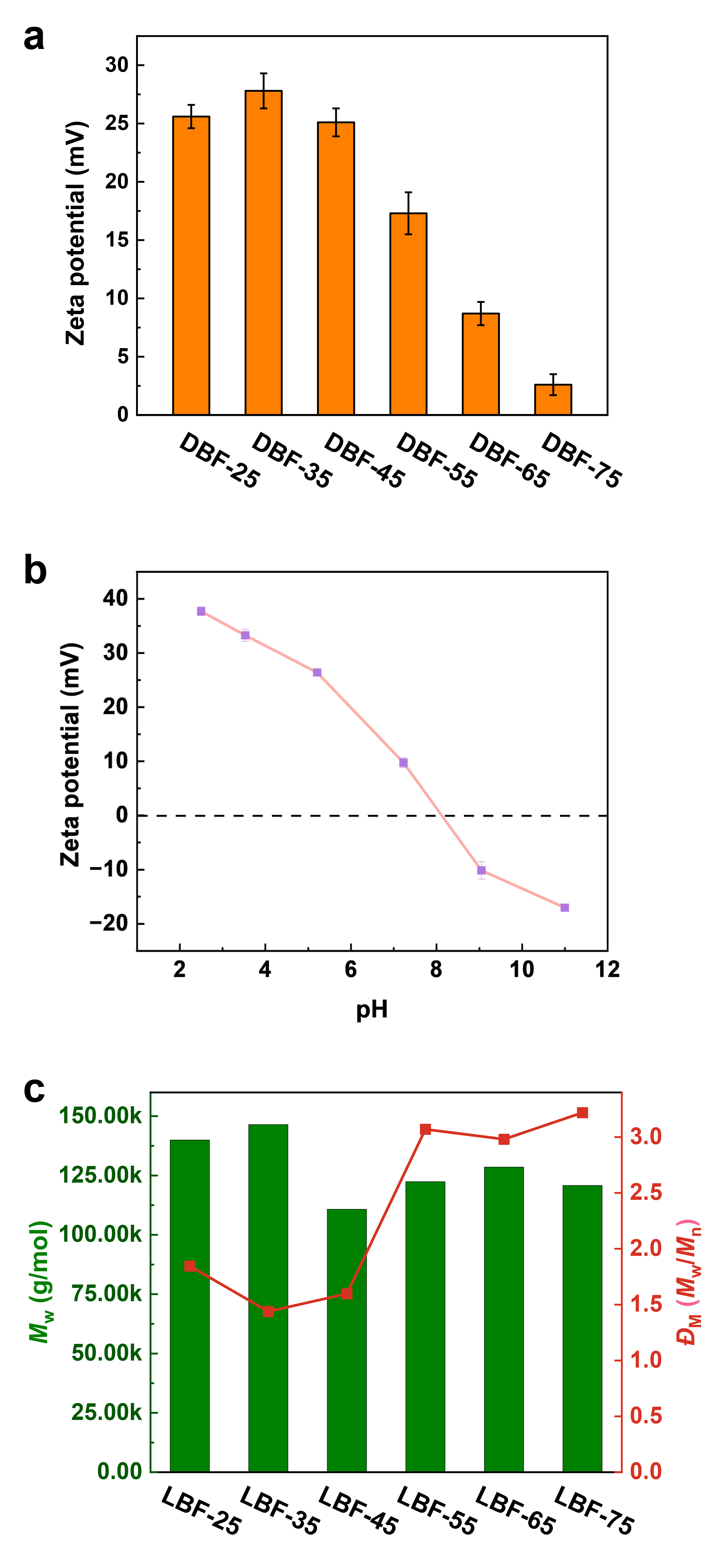


**Fig. S5.** Structural characterisation of DES-based flocculants. (a) Zeta potentials of all DES-based flocculants. (b) Zeta potentials of DBL-25 as a function of the pH value. (c) Molecular weights of all DES-based flocculants.


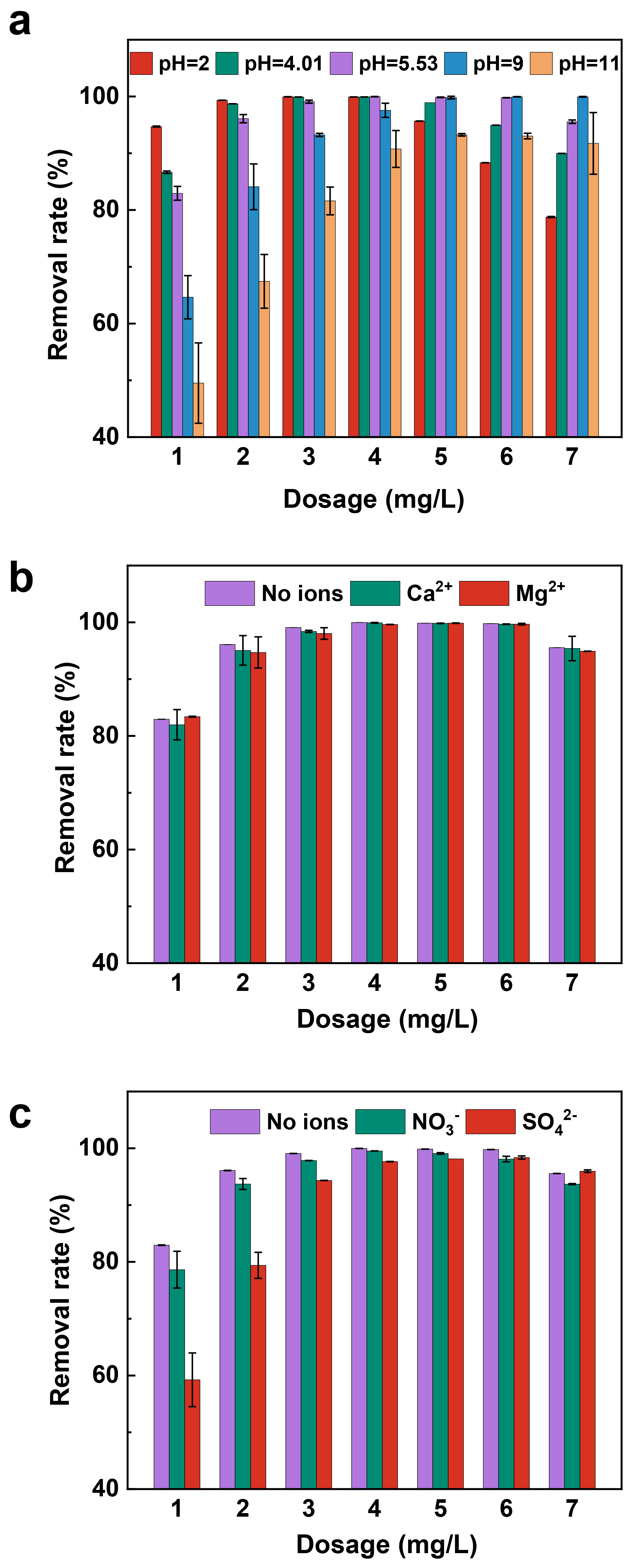


**Fig. S6.** The influence of external factors on the flocculation performance. Effects of (a) suspension pH value, (b) coexisting cation, and (c) coexisting anion on the removal rate.


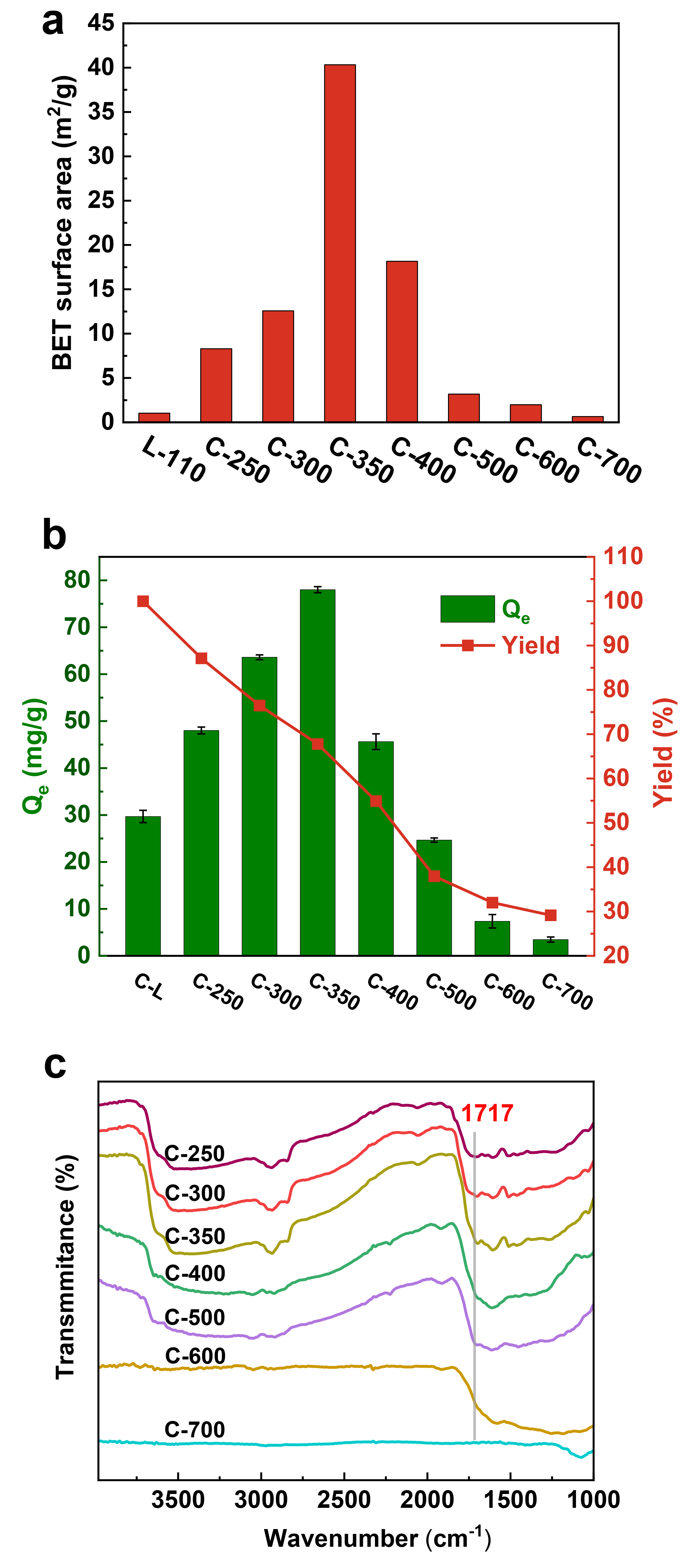


**Fig. S7.** Structural characterisation of lignin-based biochar samples. (a) The BET results of various lignin-based biochar samples. (b) Yield and corresponding adsorption capacity of lignin-based biochar samples at different calcination temperatures. (c) The FT-IR spectra of lignin-based biochar samples.


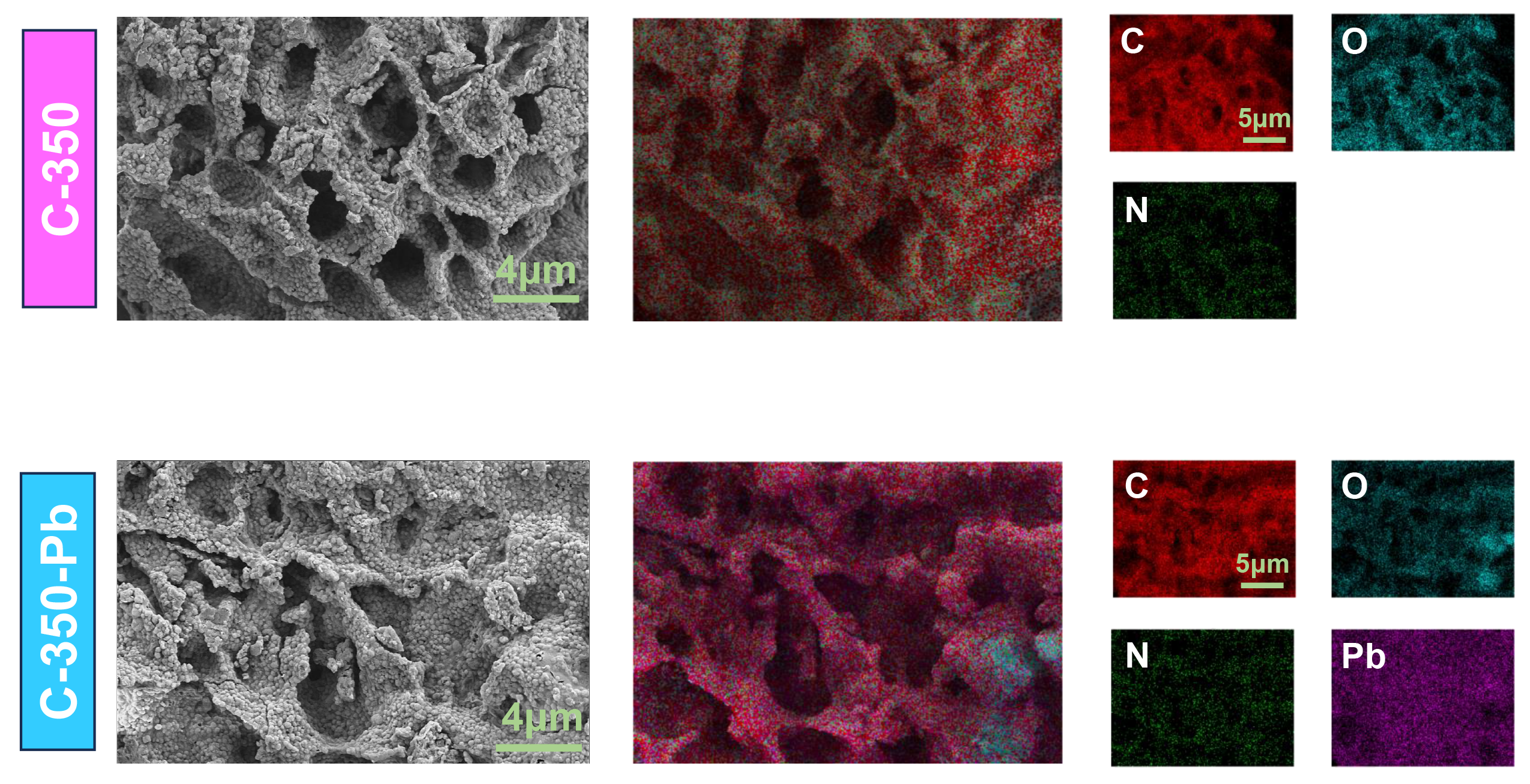


**Fig. S8.** SEM images and the corresponding elemental mapping analyses of C-350 and C-350-Pb.


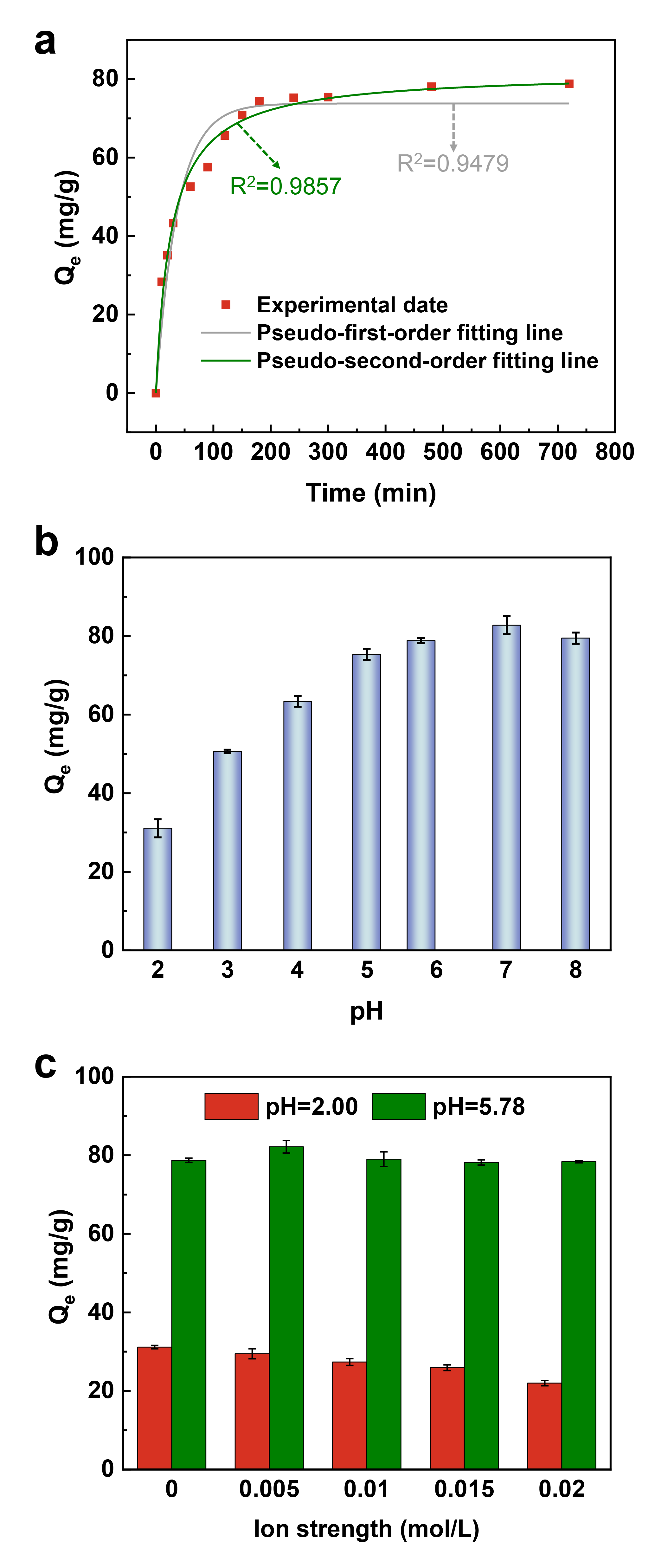


**Fig. S9.** The influence of external factors on the adsorption capacity. (a) Adsorption kinetic for Pb^2+^on C-350 and the corresponding kinetic adsorption models. The effects of wastewater pH value (b) and ionic strength (c) on the adsorption capacity of C-500.


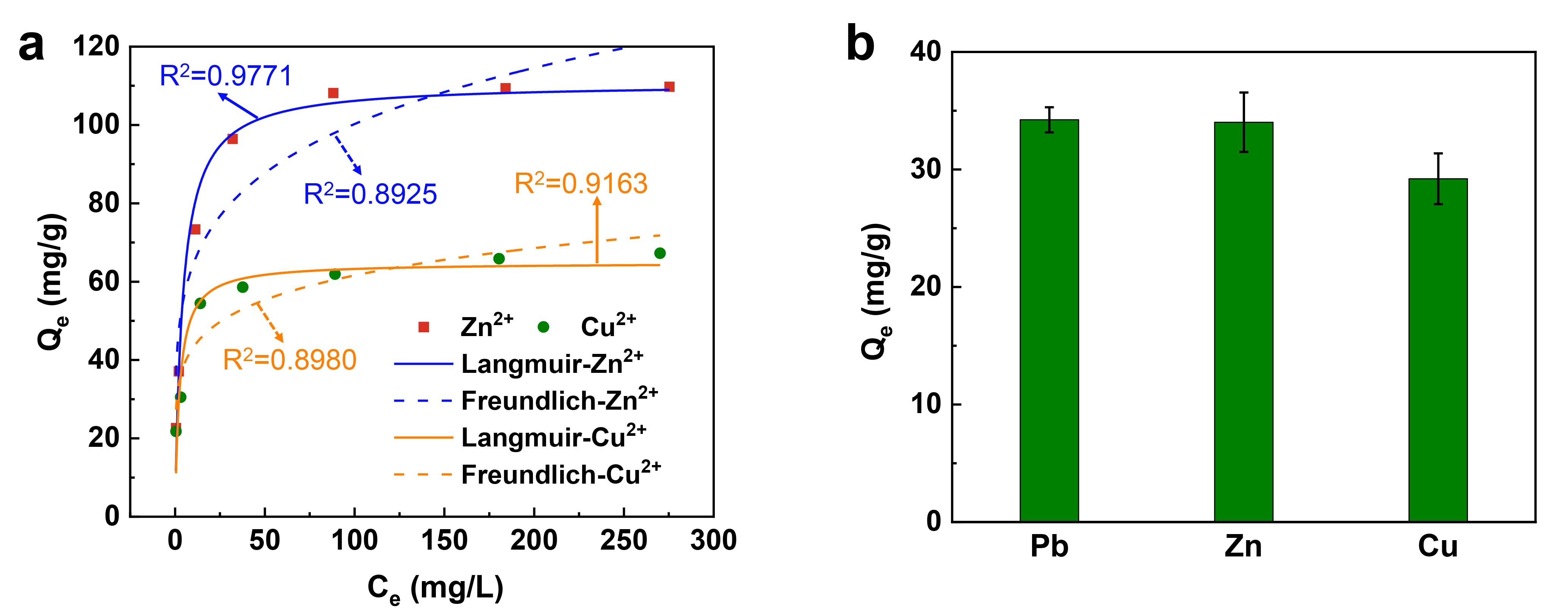


**Fig. S10.** The adsorption capacity for other heavy metal ions. (a) Adsorption isotherms for Zn^2+^ and Cu^2+^on C-350 and the corresponding isothermal adsorption models. (b) The adsorption capacity of C-500 for lead-zinc-copper mixed heavy metals simulated wastewater.


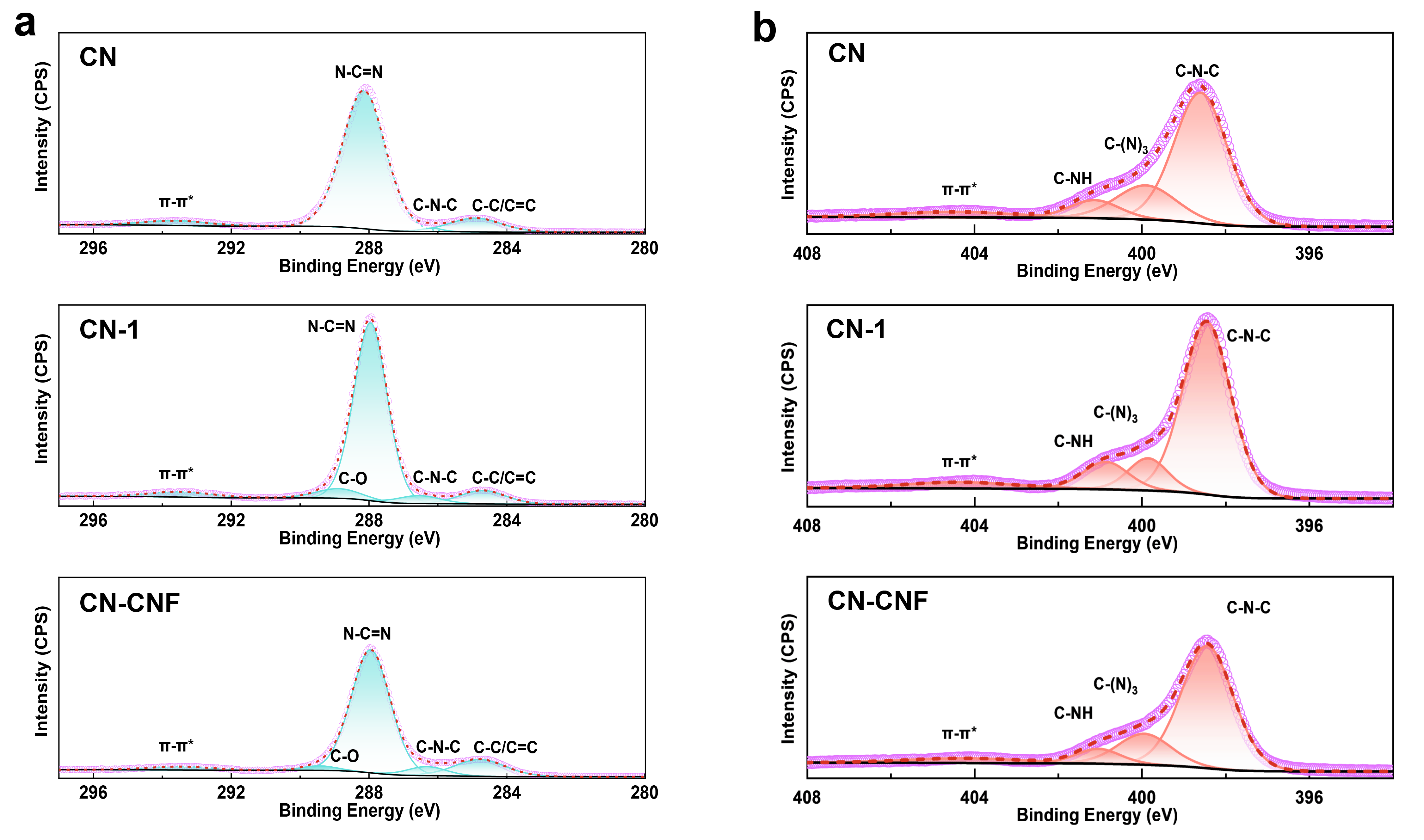


**Fig. S11**. XPS high-resolution spectra of catalysts. (a) XPS survey and high-resolution spectra of C1s. (b) XPS survey and high-resolution spectra of N1s.


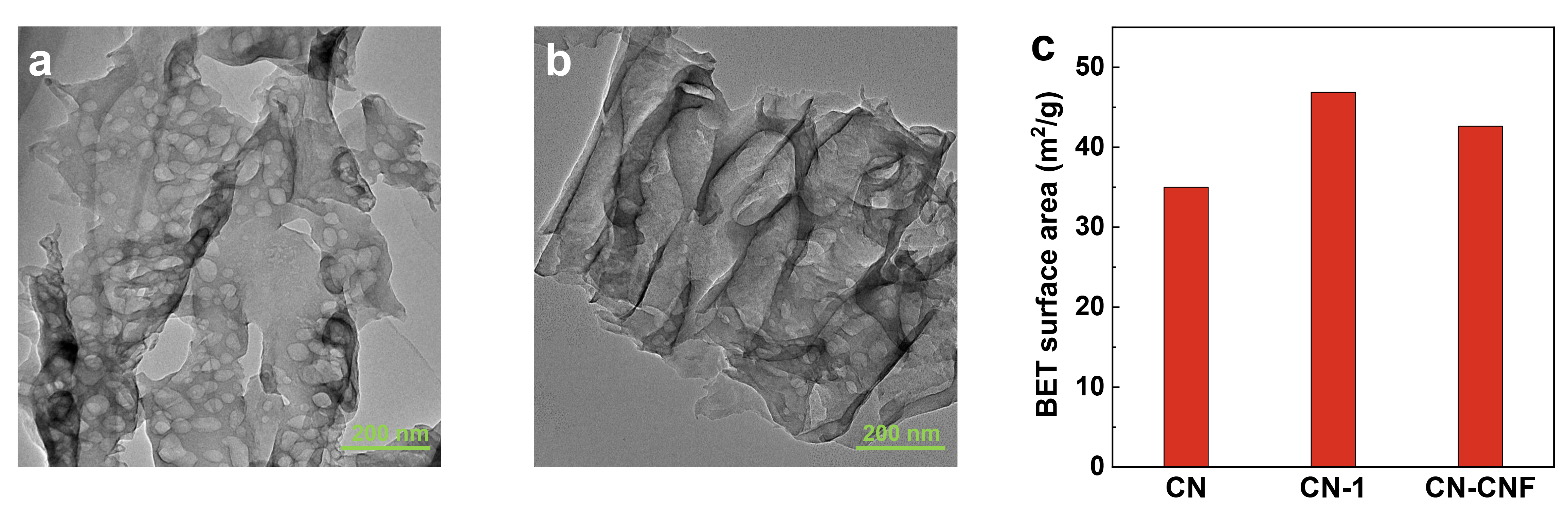


**Fig. S12.** Micromorphology and BET results of catalysts. FE-TEM images of (a) CN and (b) CN-1; (c) The BET results of CN, CN-1 and CN-CNF.


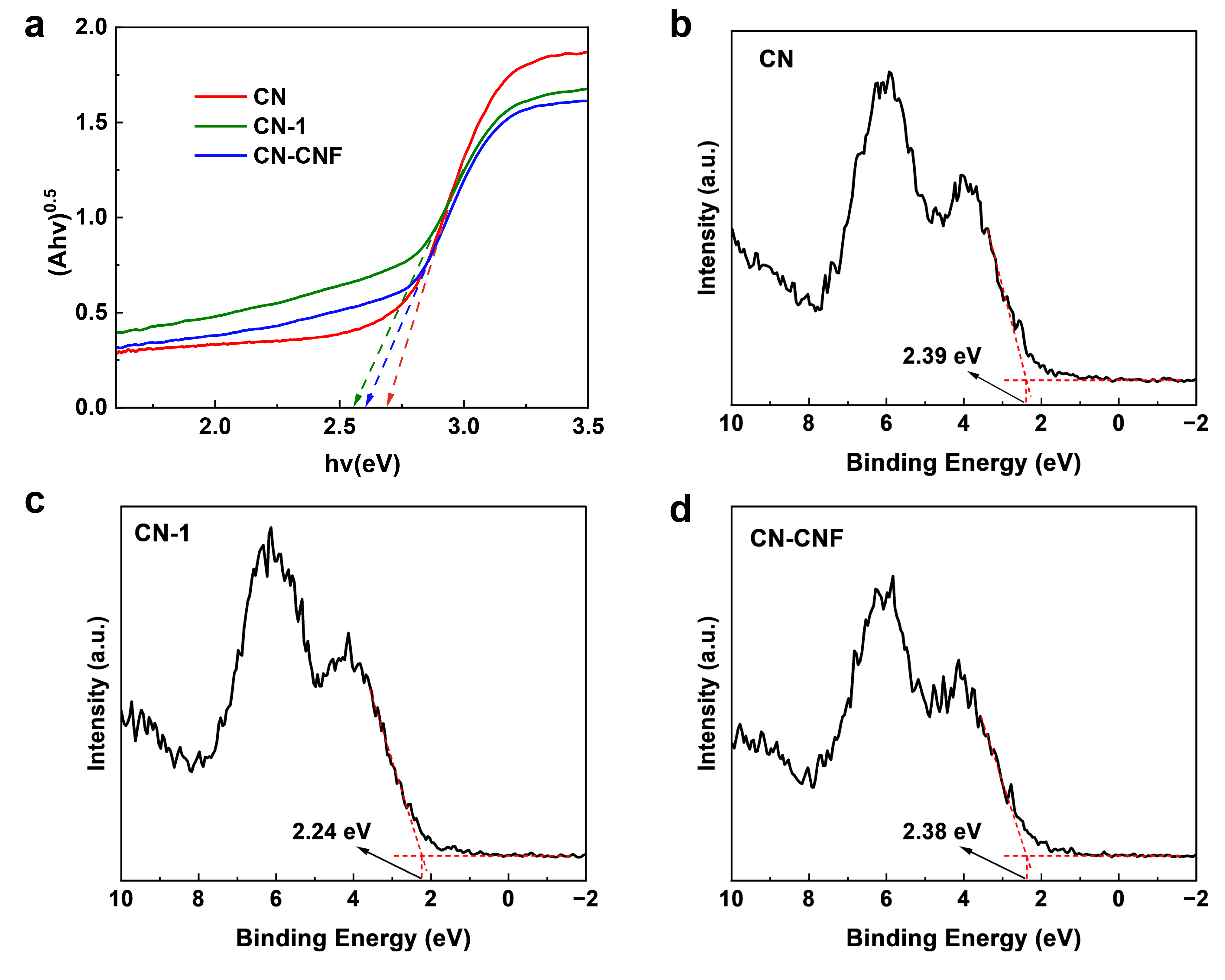


**Fig. S13.** Bandgap and VB-XPS calculations. (a) Bandgap calculation of CN, CN-1 and CN-CNF, VB-XPS calculation of (b) CN, (c) CN-1, and (d) CN-CNF.


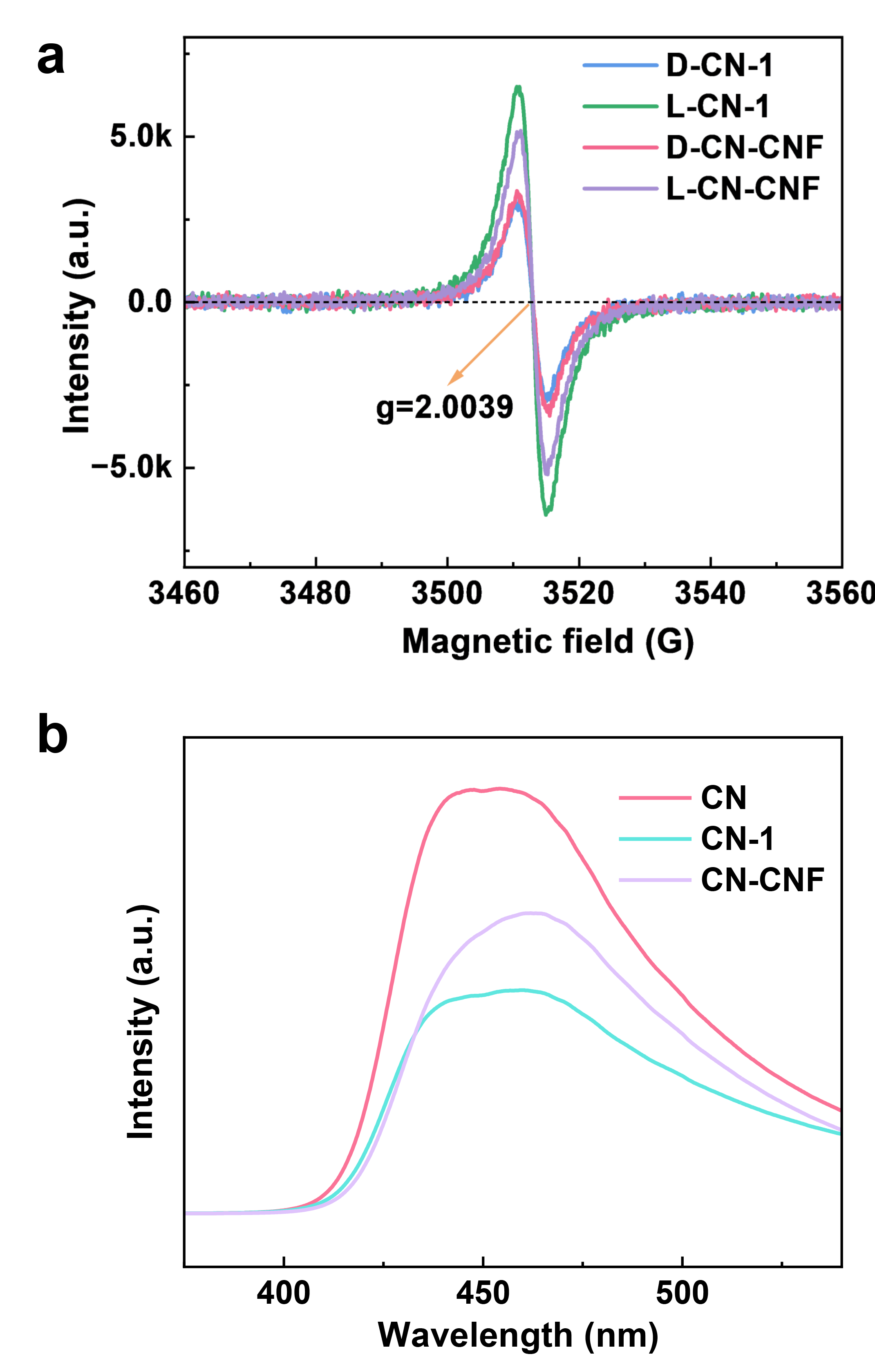


**Fig. S14.** EPR and PL spectra. (a) The EPR signals of CN-1 and CN-CNF (D: dark 10 min, L: light 10 min). (b) The PL spectra of CN, CN-1 and CN-CNF.


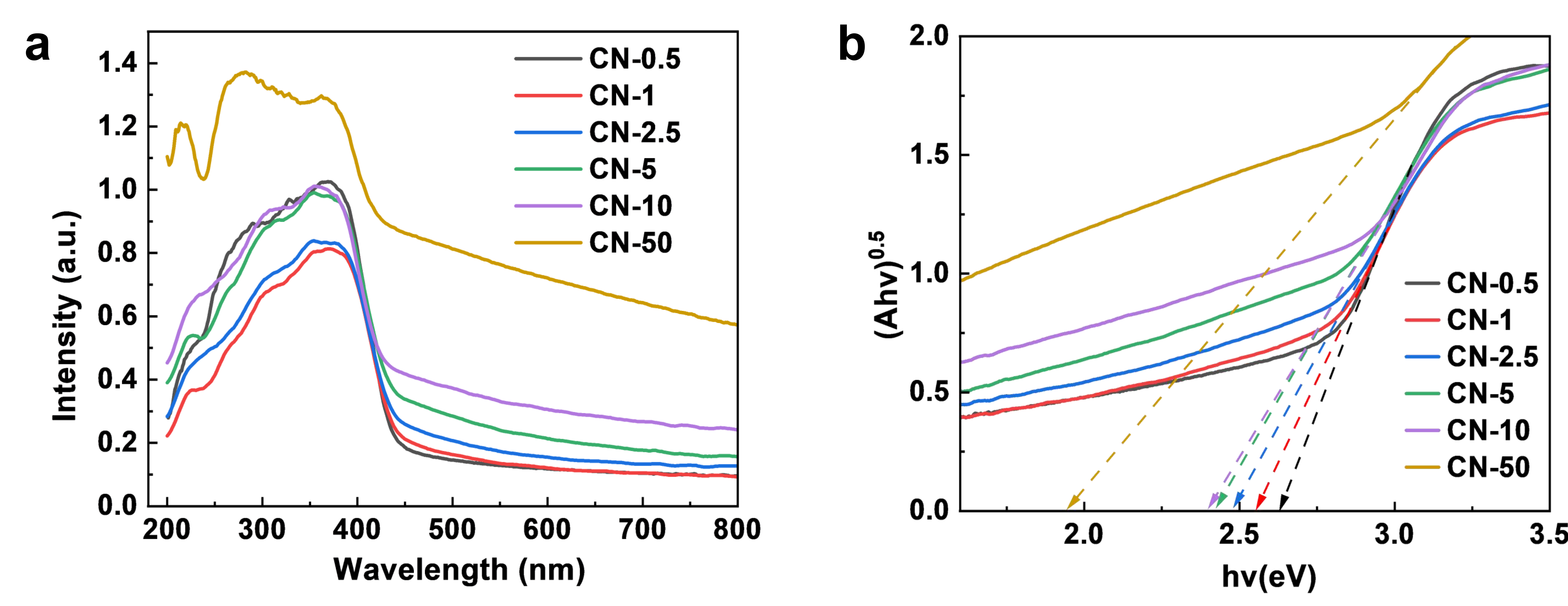


**Fig. S15.** Bandgap calculation of photocatalyst samples with different LCNF content. (a) The UV–visible absorbance spectra, and (b) bandgap calculation of carbon nitride with different LCNF additions.


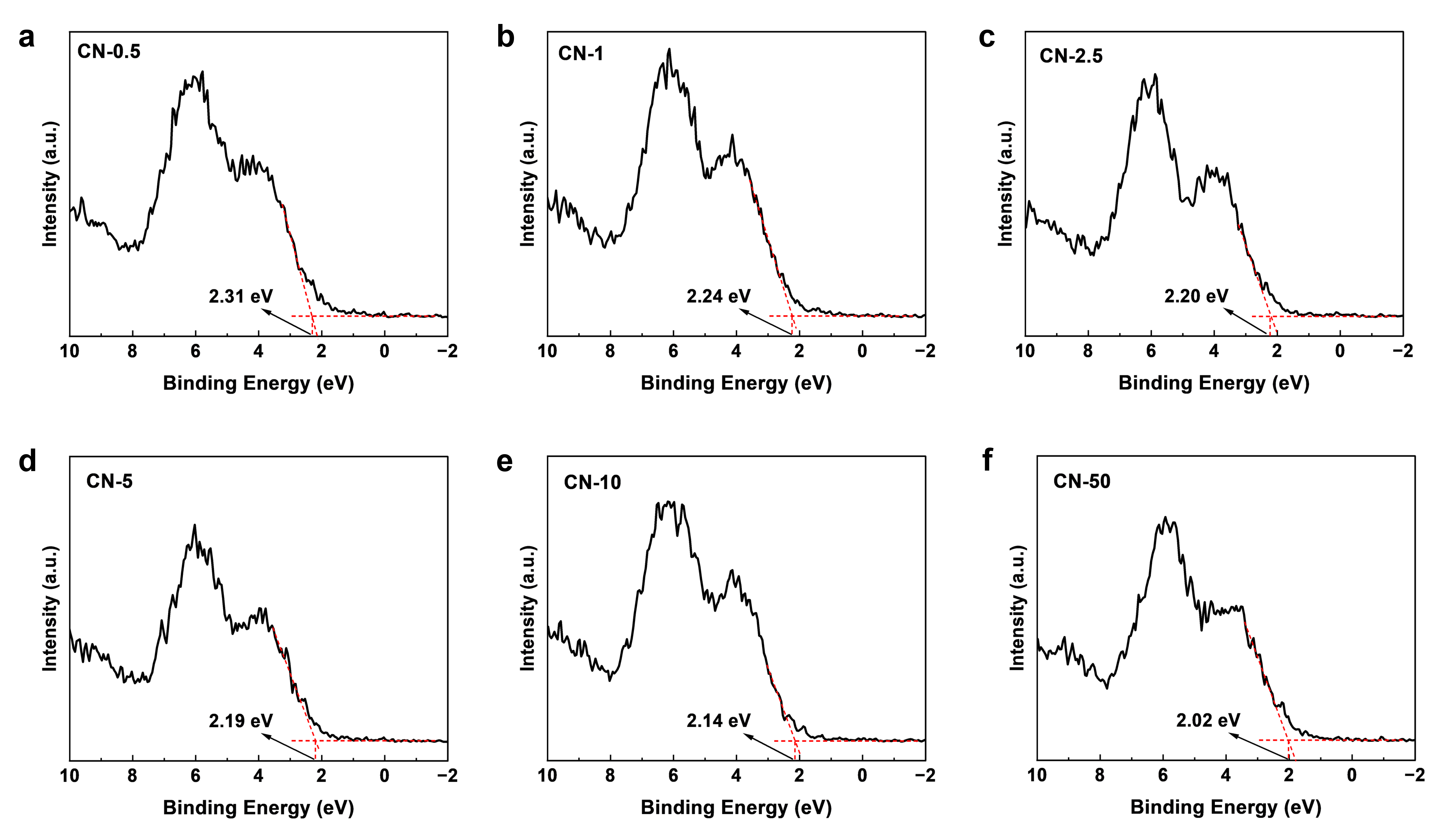


**Fig. S16.** VB-XPS calculation of photocatalyst samples with different LCNF content.


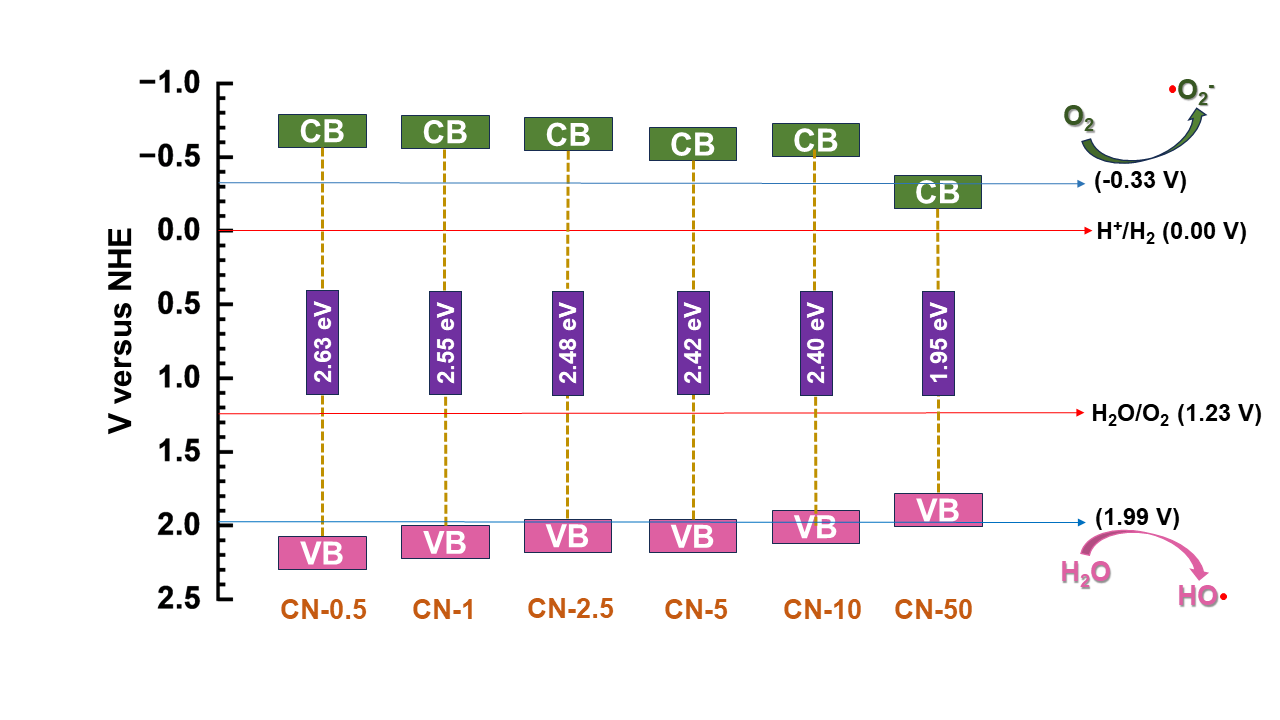


**Fig. S17.** Schematic diagram for bandgap alignment of photocatalyst samples with different LCNF content.


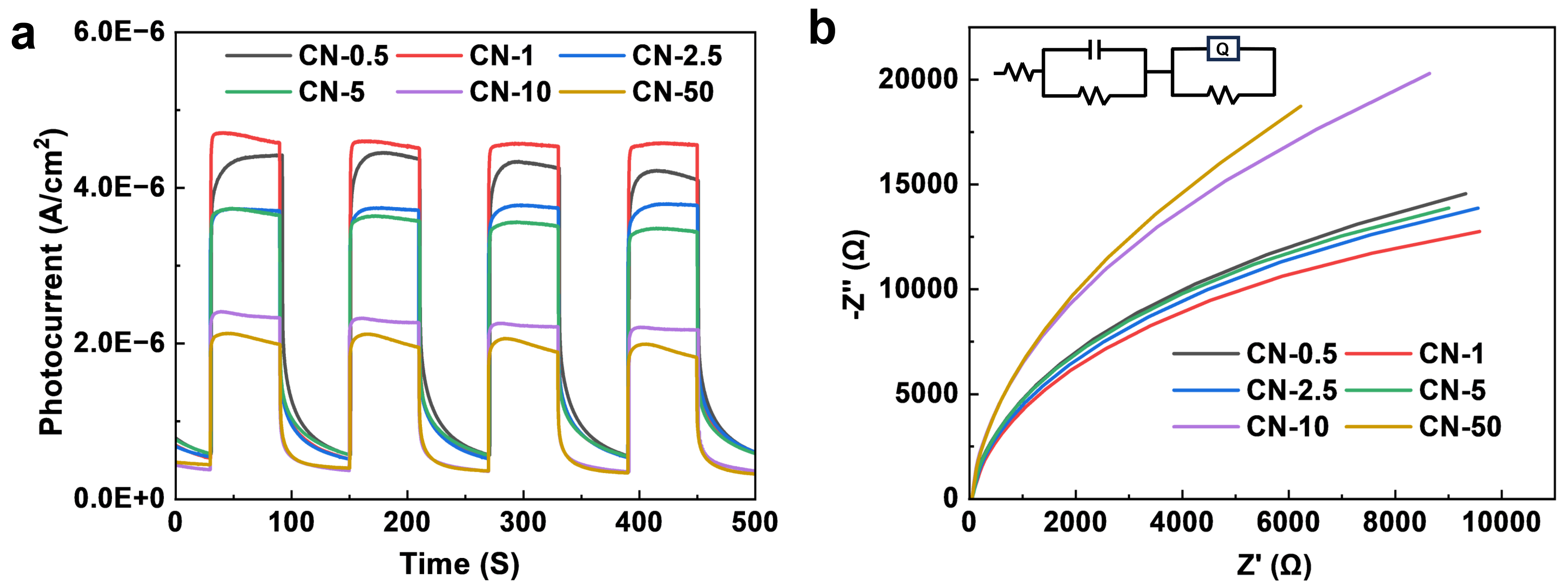


**Fig. S18.** Photochemistry spectra of photocatalyst samples with different LCNF content. The (a) Photocurrent response, and (b) Electrochemical impedance spectra of photocatalyst samples with different LCNF content.


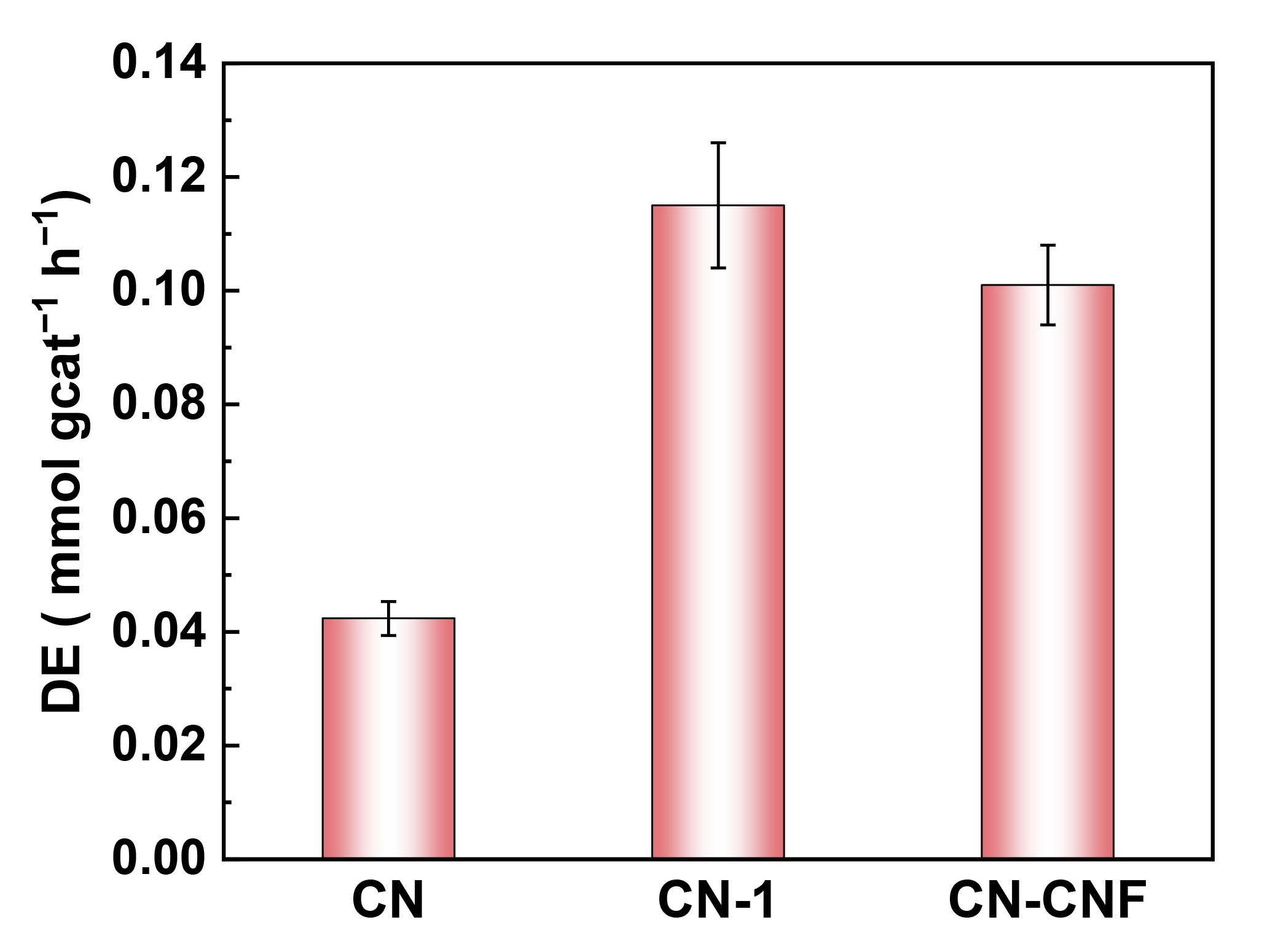


**Fig. S19.** Degradation efficiency (DE) of MO by CN-1 and CN-CNF.

**Fig. S20.** Photodegradation of MO by photocatalyst samples with different LCNF content. (a) Photodegradation, and (b) Degradation efficiency (DE) of MO by photocatalyst samples with different LCNF content.


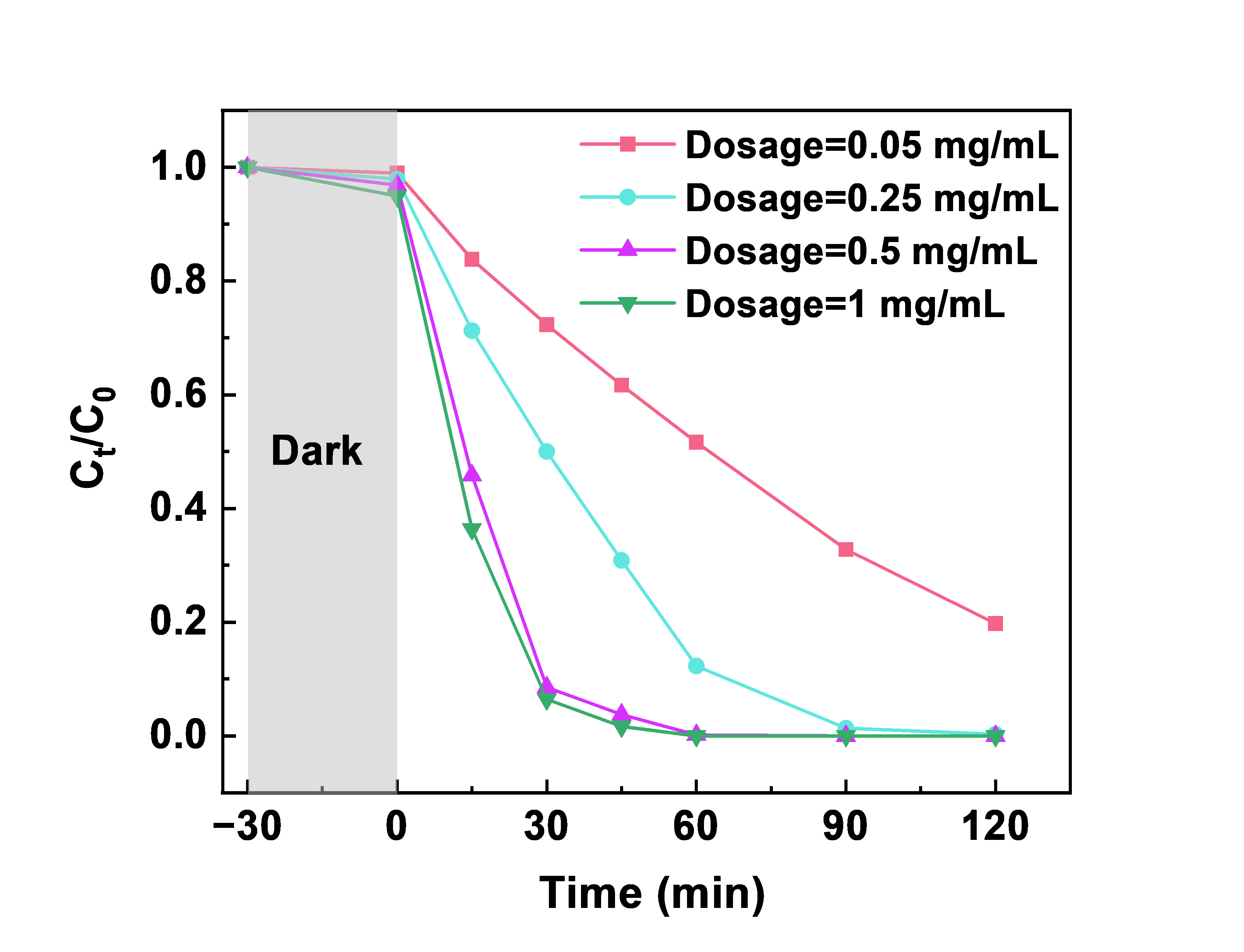


**Fig. S21.** Photodegradation of MO by CN-1 with different dosages.


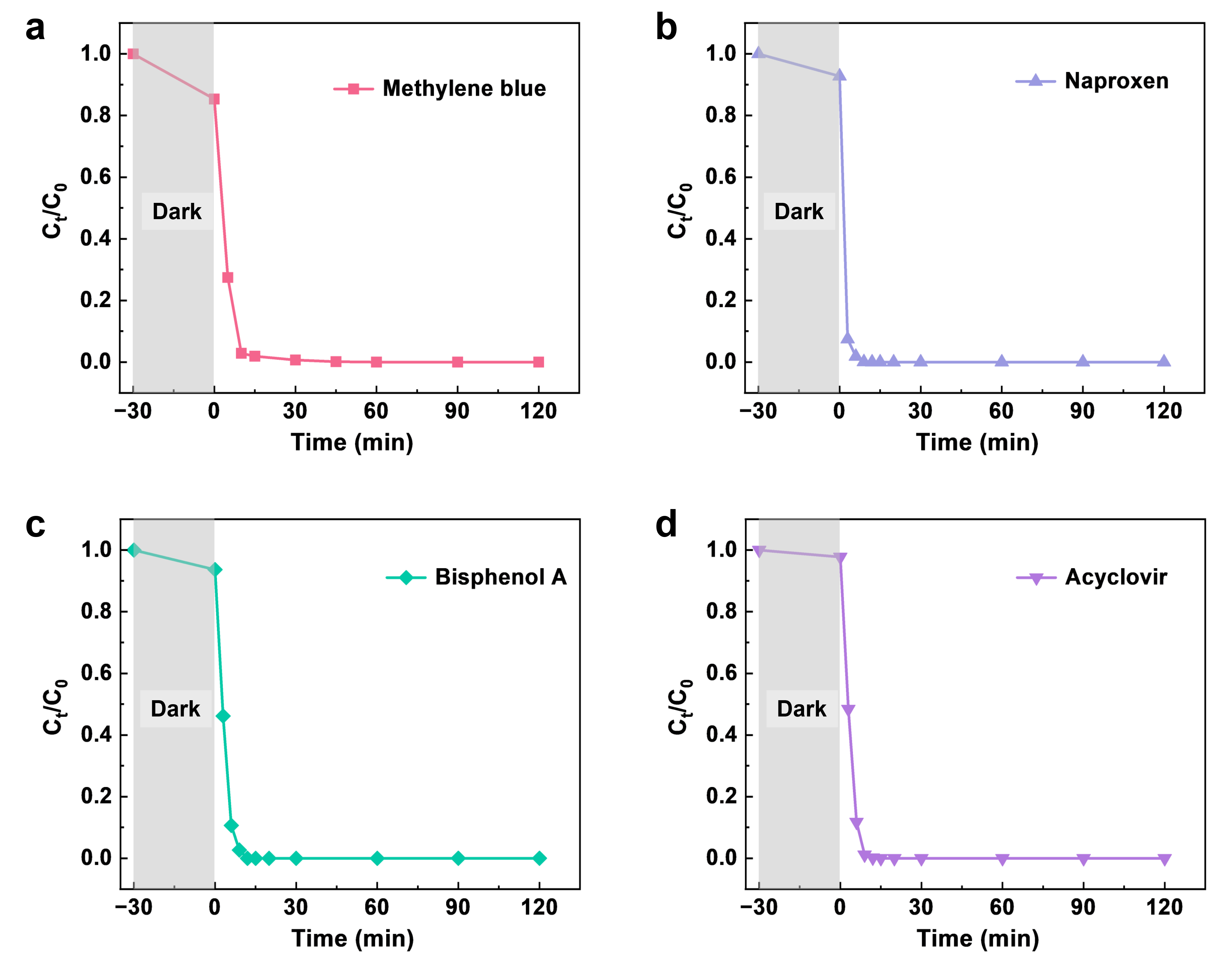


**Fig. S22.** Photodegradation of other organic pollutants. Photodegradation of (a) methylene blue, (b) naproxen, (c) bisphenol, and (d) acyclovir by CN-1 (Initial concentration: 10mg/L, 0.5 mg/mL CN-1, 300 W xenon lamp simulated sunlight, 2.5 kW/m^2^).


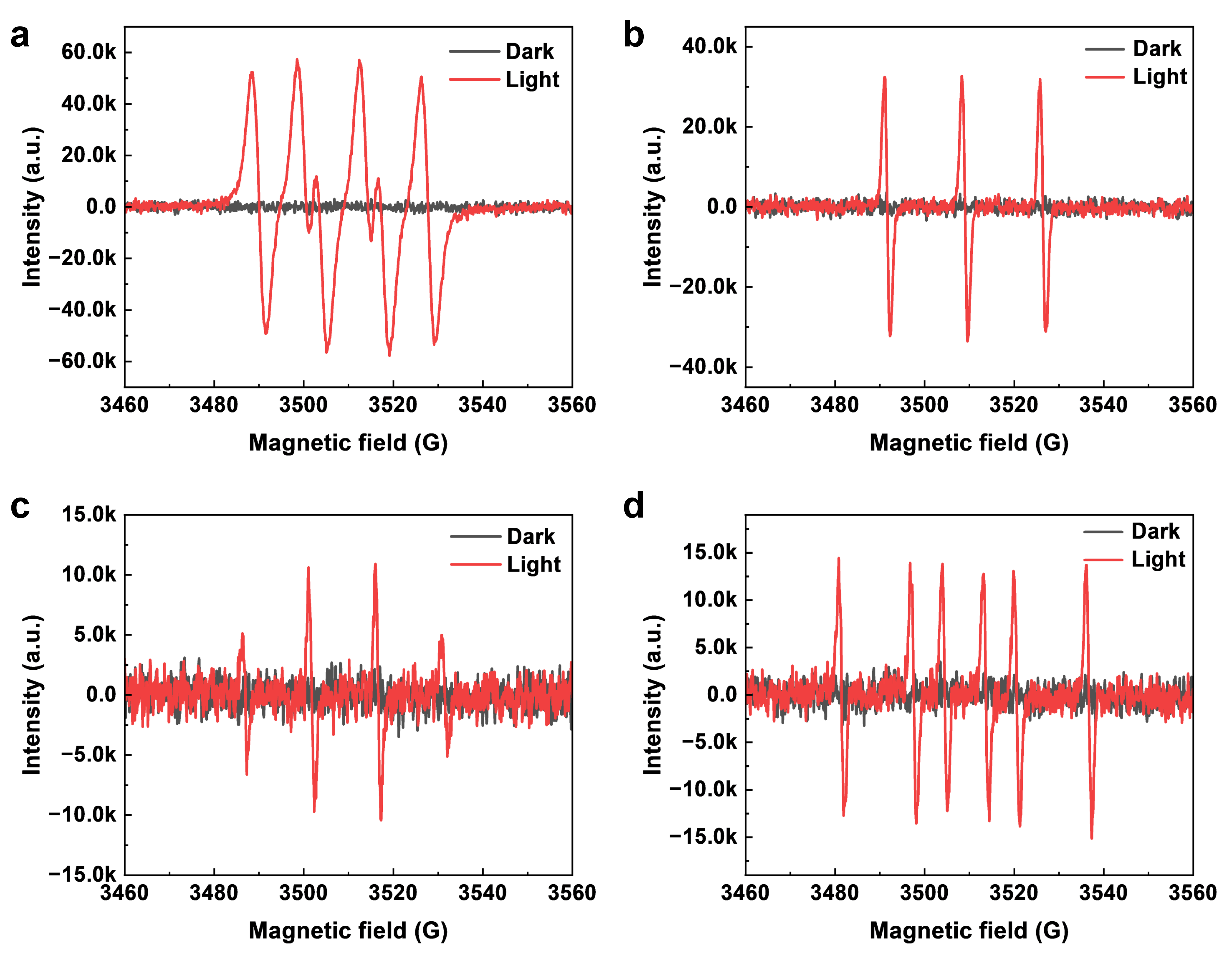


**Fig. S23.** EPR spectra of CN-1. EPR spectra of (a) DMPO/•O− 2, (b) TEMP/^1^O_2_, (c) DMPO/•O, and (d) DMPO/•R in water. (D: dark 10 min, L: light 10 min, 300 W xenon lamp simulated sunlight)


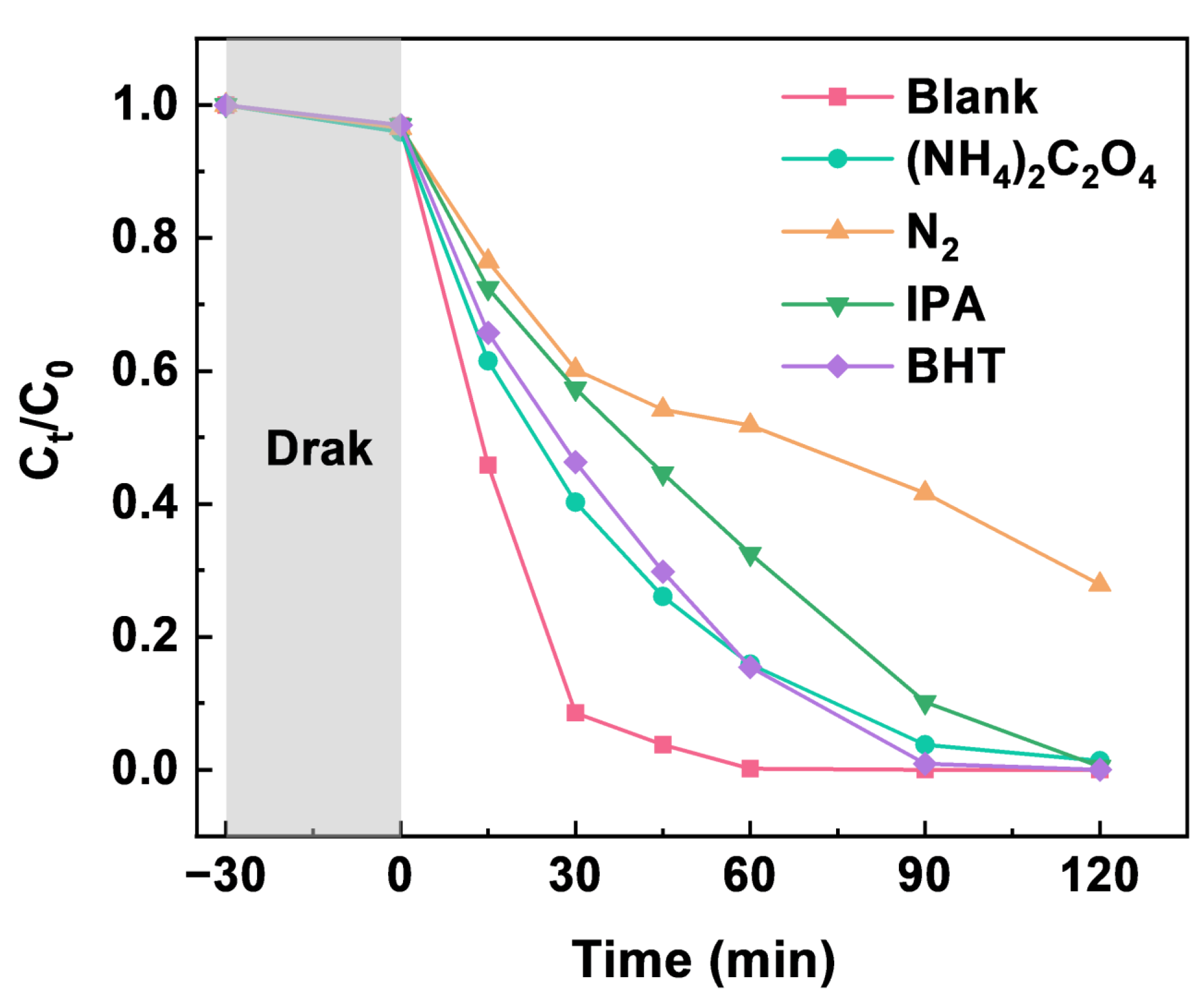


**Fig. S24.** Free radical scavenging tests. Eﬀect of various scavengers on the photocatalytic activity of CN-1 towards the degradation of MO. The photodegradation ability of CN-1 to MO decreased severely under the N_2_ atmosphere (•O− 2), followed by the addition of isopropanol (IPA, a scavenger for •OH).


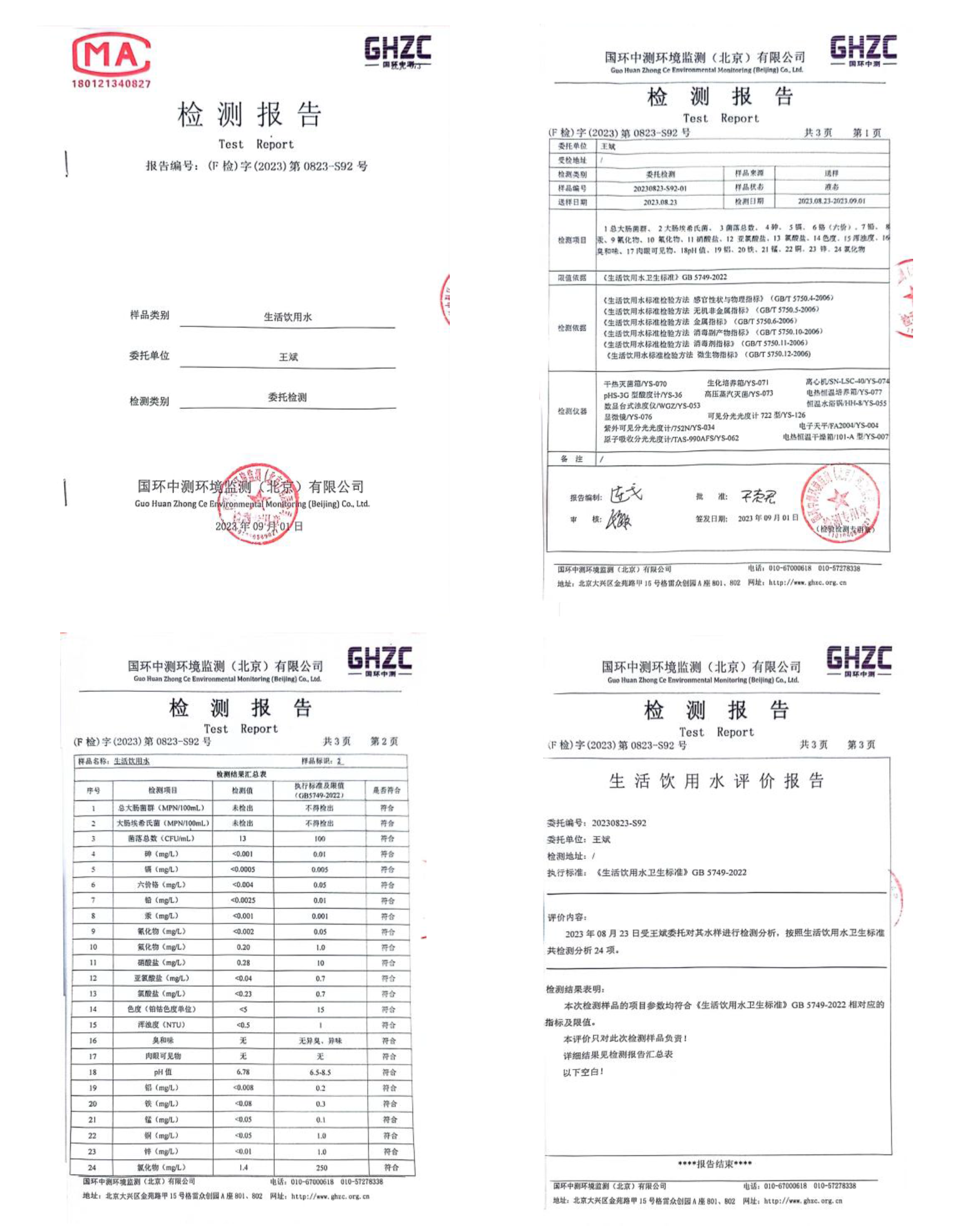


**Fig. S25.** The water quality test report of purified mineral processing wastewater.


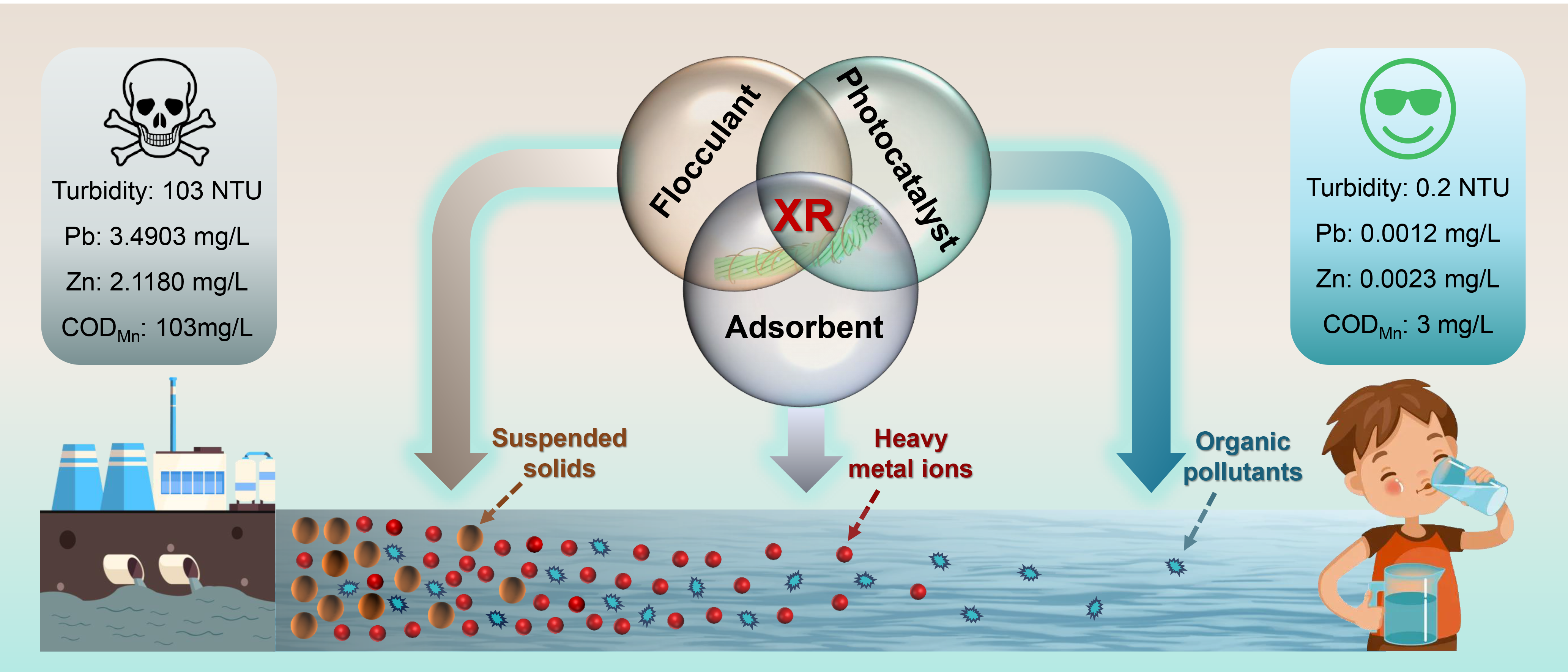


**Fig. S26.** Schematic illustration of lead-zinc mineral processing wastewater being purified into drinkable water. The lead and zinc mineral processing wastewater was purified step by step through consecutive flocculation, adsorption and photodegradation, using 5 mg/L flocculant DBL-25, 0.5 g/L adsorbent C-350 and 0.5 g/L photocatalyst CN-1 respectively, finally obtaining drinkable water. The Schematic illustration only listed the main water indicators, and the other indicators were shown in Table S4.

**
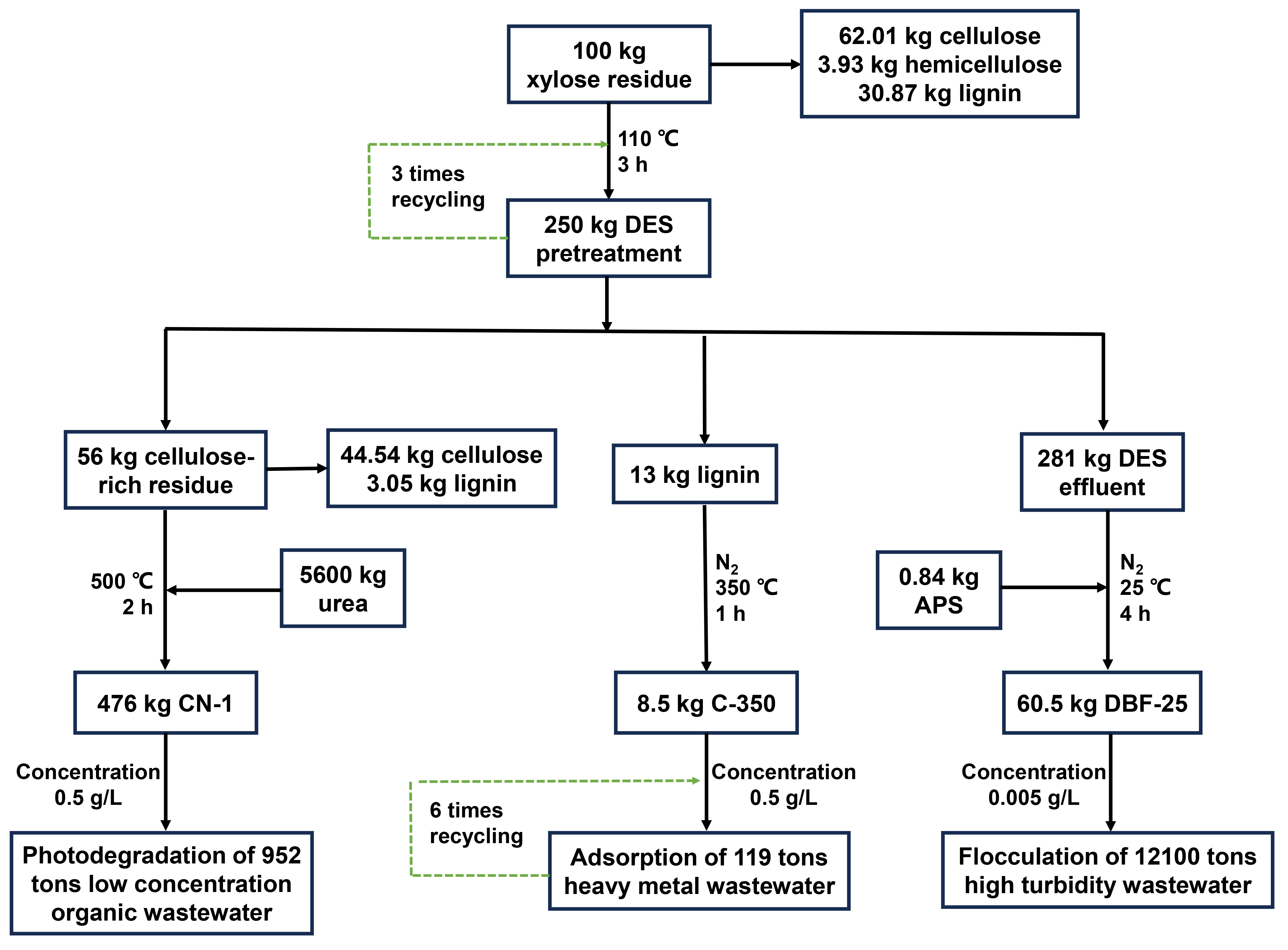
**

**Fig. S27.** Mass balance of the strategy in this study. Mass balance of DES pretreatment process, wastewater remediation materials preparation process and wastewater purification process.


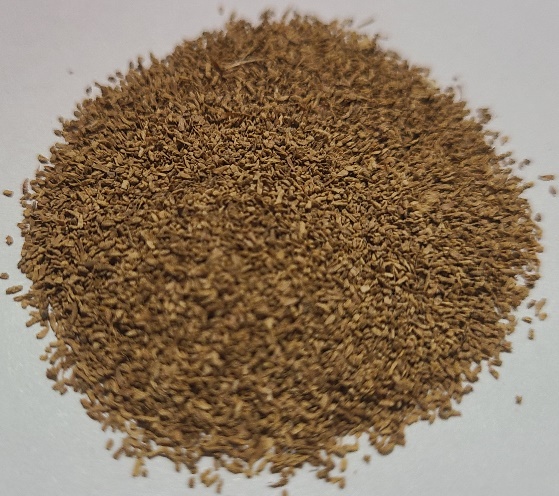


**Fig. S28.** The original picture of industrial XR.

**Table S1****.** Solid yield and chemical compositions of substrates after different pretreatment conditions (%).

| Sample | Solid yield | Hemicelluloses | Cellulose | Lignin | | Delignification ratio |
| --- | --- | --- | --- | --- | --- | --- |
|  |  |  |  | Acid soluble lignin | Klason lignin |  |
| XR | / | 3.93±0.31 | 62.01±1.06 | 2.75±0.06 | 28.12±0.21 | / |
| R-70 | 63.85±0.31 | N.D. | 78.81±0.65 | 0.83±0.04 | 17.58±0.13 | 61.93±1.35 |
| R-90 | 58.85±0.27 | N.D. | 82.19±0.73 | 0.65±0.07 | 13.03±0.24 | 73.92±2.76 |
| R-110 | 56.48±0.32 | N.D. | 92.80±0.71 | 0.44±0.03 | 5.91±0.19 | 88.37±1.52 |
| R-130 | 53.71±0.46 | N.D. | 88.65±0.76 | 0.47±0.03 | 9.52±0.25 | 82.62±1.83 |

**Table S2.** Quantification of lignin component by quantitative ^31^P NMR spectroscopy (mmol/g) and the molecular weights of lignin samples (g/mol).

| Sample | Aliphatic OH | Syringyl OH | Guaiacyl OH | | Hydroxyphenyl OH | Carboxylic group | Total phenolic OH^c^ | *M*n | *M*w | *Ð*_M_ |
| --- | --- | --- | --- | --- | --- | --- | --- | --- | --- | --- |
|  |  |  | C^a^ | NC^b^ |  |  |  |  |  |  |
| DEL | 2.79 | 0.46 | 0.14 | 0.57 | 0.74 | 0.15 | 1.91 | 3970 | 7720 | 1.94 |
| L-70 | 1.81 | 0.59 | 0.20 | 0.83 | 1.20 | 0.51 | 2.83 | 1170 | 3280 | 2.80 |
| L-90 | 1.66 | 0.64 | 0.21 | 0.82 | 1.13 | 0.66 | 2.80 | 1050 | 2520 | 2.41 |
| L-110 | 1.37 | 0.78 | 0.25 | 0.86 | 1.01 | 1.06 | 2.90 | 920 | 1900 | 2.07 |
| L-130 | 0.95 | 0.62 | 0.20 | 0.68 | 0.82 | 1.37 | 2.32 | 770 | 1600 | 2.07 |

^a^ C, condensed.

^b^ NC, non-condensed.

^c^ Total phenolic OH, the sum of syringyl OH, guaiacyl OH and Hydroxyphenyl OH.

**Table S3.** The isothermal adsorption model parameters of C-350.

| Heavy metal | Langmuir | | |  | Freundlich | | |
| --- | --- | --- | --- | --- | --- | --- | --- |
|  | Q_m_ (mg/g) | k_L_ | R^2^ |  | k_F_ | n | R^2^ |
| Pd^2+^ | 143.21 | 0.1333 | 0.9822 |  | 42.3706 | 0.2375 | 0.9446 |
| Zn^2+^ | 110.64 | 0.2366 | 0.9771 |  | 41.2450 | 0.1928 | 0.8925 |
| Cu^2+^ | 64.84 | 0.3988 | 0.9163 |  | 30.2408 | 0.1545 | 0.8980 |

**Table S4.** Results of various parameters in wastewater and the strictest drinking water standards.

| Parameters | Raw wastewater | FW | FAW | FAPW | Standards ^a^ |
| --- | --- | --- | --- | --- | --- |
| Turbidity (NTU) | 103 | 0.4 | 0.2 | 0.2 | 1 |
| Pb (mg/L) | 3.4903 | 3.1125 | 0.0051 | 0.0012 | 0.01 |
| Zn (mg/L) | 2.1180 | 1.9275 | 0.0076 | 0.0023 | 1 |
| Cu (mg/L) | 0.9062 | 0.8623 | 0.0008 | 0 | 1 |
| COD_Mn_ | 103 | 96 | 77 | 3 | 5 |
| Total Coliform Bacteria (MPN/100 mL) | N.D. | / | / | N.D. | N.D. |
| Escherichia coli  (MPN/100 mL) | N.D. | / | / | N.D. | N.D. |
| Total colony number  (CFU/ mL) | 48 | / | / | 13 | 100 |
| As (mg/L) | <0.001 | / | / | <0.001 | 0.01 |
| Cd (mg/L) | <0.0005 | / | / | <0.0005 | 0.005 |
| Cr^6+^ (mg/L) | <0.004 | / | / | <0.004 | 0.05 |
| Hg (mg/L) | <0.001 | / | / | <0.001 | 0.001 |
| Cyanide (mg/L) | <0.002 | / | / | <0.002 | 0.05 |
| Fluoride (mg/L) | 0.52 | / | / | 0.20 | 1 |
| Nitrate (mg/L) | 4.05 | / | / | 0.28 | 10 |
| Hypochlorite (mg/L) | <0.04 | / | / | <0.04 | 0.7 |
| Chlorate (mg/L) | <0.23 | / | / | <0.23 | 0.7 |
| Odor | Yes | / | / | N.D. | N.D. |
| Visible particles | Yes | / | / | N.D. | N.D. |
| pH value | 8.31 | / | / | 7.78 | 6.5-8.5 |
| Al (mg/L) | <0.008 | / | / | <0.008 | 0.2 |
| Fe (mg/L) | <0.08 | / | / | <0.08 | 0.2 |
| Mn (mg/L) | <0.05 | / | / | <0.05 | 0.05 |
| Chloride | 15.0 | / | / | 1.4 | 250 |

^a^, The strictest standards for drinkable water quality from the World Health Organization (http://apps.who.int/iris/), EU Drinking Water Directive (98/83/EC), U.S. Environmental Protection Agency (https://www.epa.gov/), and China's national standards (GB 5749-2022).

**Reference**

1 Sluiter A, Hames B, Ruiz R, Scarlata C, Sluiter J, Templeton D, Crocker DJLap. Determination of structural carbohydrates and lignin in biomass. *Laboratory analytical procedure*. 2008; 1617(1):1-16.

2 Argyropoulos DS. ^31^P NMR in wood chemistry: a review of recent progress. *Res. Chem. Intermediat*. 1995; 21(3-5):373-395.

3 Meng X, Crestini C, Ben H, Hao N, Pu Y, Ragauskas AJ, Argyropoulos DS. Determination of hydroxyl groups in biorefinery resources via quantitative ^31^P NMR spectroscopy. *Nat Protoc*. 2019; 14(9):2627-2647.

4 Shen XJ, Wang B, Huang PL, Wen JL, Sun RC. Effects of aluminum chloride-catalyzed hydrothermal pretreatment on the structural characteristics of lignin and enzymatic hydrolysis. *Bioresource Technol*. 2016; 206:57-64.

5 Li YX, Shang HR, Cao YN, Yang CH, Feng YJ, Yu YL. High performance removal of sulfamethoxazole using large specific area of biochar derived from corncob xylose residue. *Biochar*. 2022; 4(1):11.

6 Ma CY, Sun Q, Zuo C, Xu LH, Sun SN, Wen JL, Yuan TQ. Efficient fractionation and targeted valorization of industrial xylose residue by synergistic and mild alkaline deep eutectic solvent‑hydrogen peroxide pretreatment. *Fuel Process Technol*. 2023; 241:107591.

7 Mendoza DJ, Browne C, Raghuwanshi VS, Simon GP, Garnier G. One-shot TEMPO-periodate oxidation of native cellulose. *Carbohyd Polym*. 2019; 226:115292.

8 Rencoret J, Prinsen P, Gutiérrez A, Martínez AT, del Río JC. Isolation and structural characterization of the milled wood lignin, dioxane lignin, and cellulolytic lignin preparations from brewer's spent grain. *J Agr Food Chem*. 2015; 63(2):603-613.

9 Shen XJ, Chen T, Wang HM, Mei Q, Yue F, Sun S, Wen JL, Yuan TQ, Sun R-C. Structural and morphological transformations of lignin macromolecules during bio-based deep eutectic solvent (DES) pretreatment. *ACS Sustainable Chem Eng*. 2019; 8(5):2130-2137.

10 Nimbalkar MN, Bhat BR. Simultaneous adsorption of methylene blue and heavy metals from water using Zr-MOF having free carboxylic group. *J Environ Chem Eng*. 2021; 9(5):106216.

11 Chang XJ, Li XS, Ge SJ, Tan GL, Wang ZD, Ma YF, Niu L, Li QH. Insight into the oil removal mechanism of quaternary ammonium ionic liquid microemulsions for oily sludge treatment. *Sustain Energ Fuels*. 2023; 7(9):2153-2162.

12 Wang B, Wang H-M, Sun D, Yuan TQ, Song GY, Shi Q, Zheng L, Wang SF, Sun RC. Chemosynthesis, characterization and application of lignin-based ﬂocculants with tunable performance prepared by short-wavelength ultraviolet initiation. *Ind Crop Prod*. 2020; 157:112897.

13 Faix O. Classification of lignins from different botanical origins by ft-ir spectroscopy. *Holzforschung*, 1991; 45(s1):21-28.

14 Cao JL, He GH, Ning XQ, Chen XH, Fan LH, Yang M, Yin YH, Cai WQ. Preparation and properties of O-chitosan quaternary ammonium salt/polyvinyl alcohol/graphene oxide dual self-healing hydrogel. *Carbohyd Polym*. 2022; 287:119318.

15 Wang B, Sun Z, Lam SS, Sonne C, Yuan TQ, Sun RC. A scalable and simple lignin-based polymer for ultra-efficient flocculation and sterilization. *Sep Purif Technol*. 2022; 292:120960.

16 Yang R, Li HJ, Huang M, Yang H, Li AM. A review on chitosan-based flocculants and their applications in water treatment. *Water Res*. 2016; 95:59-89.

17 Urry DW. Neutral sites for calcium ion binding to elastin and collagen: a charge neutralization theory for calcification and its relationship to atherosclerosis. *P Natl Acad Sci. USA*., 1971; 68(4):810-814.

18 Li SX, Hu TY, Xu YZ, Wang JY, Chu RY, Yin ZH, Mo F, Zhu LD. A review on flocculation as an efficient method to harvest energy microalgae: mechanisms, performances, influencing factors and perspectives. *Renew Sust Energ Rev*. 2020; 131:110005.

19 Chen JC, Kazzaz AE, AlipoorMazandarani, N., Feizi, Z. H. & Fatehi, P. Production of flocculants, adsorbents, and dispersants from lignin. *Molecules*. 2018; 23(4):868.

20 Wang KX, Wei TT, Li YN, He L, Lv Y, Chen L, Ahmad A, Xu YS, Shi YL. Flocculation-to-adsorption transition of novel salt-responsive polyelectrolyte for recycling of highly polluted saline textile effluents. *Chem Eng J*. 2021; 413:127410.

21 Huang M, Wang YW, Cai J, Bai JF, Yang H, Li AM. Preparation of dual-function starch-based flocculants for the simultaneous removal of turbidity and inhibition of in water. *Water Res*. 2016; 98:128-137.

22 Yip KV, Xu MH, Li CZ, Jiang SP, Wu HW. Biochar as a fuel: 3. mechanistic understanding on biochar thermal annealing at mild temperatures and its effect on biochar reactivity. *Energ Fuel*. 2011; 25(1):406-414.

23 Chen YD, Bai SW, Li RX, Su GY, Duan XG, Wang SB, Ren NQ, Ho SH. Magnetic biochar catalysts from anaerobic digested sludge: Production, application and environment impact. *Environ Int*. 2019; 126:302-308.

24 Oliveira ON, Caseli L, Ariga K. The past and the future of langmuir and langmuir–blodgett films. *Chem Rev*. 2022; 122(6):6459-6513.

25 Zhang Z, Chen Y, Wang D, Yu D, Wu C. Lignin-based adsorbents for heavy metals. *Ind Crop Prod*. 2023; 193:116119.

26 Sun Y, Wang T, Sun X, Bai L, Han C, Zhang P. The potential of biochar and lignin-based adsorbents for wastewater treatment: comparison, mechanism, and application—a review. *Ind Crop Prod*. 2021; 166:113473.

27 Li N, An X, Xiao X, An W, Zhang Q. Recent advances in the treatment of lignin in papermaking wastewater. *World J Microb Biot*. 2022; 38(7):143-173.

28 Hu XY, Yan LL, Wang YM, Xu M. Self-assembly of binary oppositely charged polysaccharides into polyelectrolyte complex hydrogel film for facile and efficient Pb removal. *Chem Eng J*. 2020; 388:124189.

29 Liang R-h, Li Y, Huang L, Wang X-d, Hu X-x, Liu C-m, Chen M-s, Chen J. Pb^2+^ adsorption by ethylenediamine-modified pectins and their adsorption mechanisms. *Carbohyd Polym*. 2020; 234:115911.

30 Cui XQ, Fang SY, Yao YQ, Li TQ, Ni QJ, Yang XE, He ZL. Potential mechanisms of cadmium removal from aqueous solution by derived biochar. *Sci Total Environ*. 2016; 562:517-525.

31 Wu FF, Chen L, Hu P, Wang YX, Deng J, Mi BB. Industrial alkali lignin-derived biochar as highly efficient and low-cost adsorption material for Pb(II) from aquatic environment. *Bioresource Technol*. 2021; 322:124539.

32 Chu YC, Lin TJ, Lin YR, Chiu WL, Nguyen BS, Hu C. Influence of P,S,O-doping on g-C_3_N_4_ for hydrogel formation and photocatalysis: an experimental and theoretical study. *Carbon*. 2020; 169:338-348.

33 Mohamed MA, M. Zain MF, Jeffery Minggu L, Kassim MB, Saidina Amin NA, W. Salleh WN, Salehmin MNI, Md Nasir MF, Mohd Hir ZA. Constructing bio-templated 3D porous microtubular C-doped g-C_3_N_4_ with tunable band structure and enhanced charge carrier separation. *Appl Catal B-Environ*. 2018; 236:265-279.

34 Li J, Zhang K, Zhao Y, Wang C, Wang L, Wang L, Liao M, Ye L, Zhang Y, Gao Y, Wang B, Peng H. High‐efficiency and stable Li−CO_2_ battery enabled by carbon nanotube/carbon nitride heterostructured photocathode. *Angew Chem Int Edit*. 2021; 61(4):e202114612.

35 Tay Q, Kanhere P, Ng CF, Chen S, Chakraborty S, Huan ACH, Sum TC, Ahuja R, Chen Z. Defect engineered g-C_3_N_4_ for efficient visible light photocatalytic hydrogen production. *Chem Mater*. 2015; 27(14):4930-4933.

36 Deng PH, Li HY, Wang ZD, Hou Y. Enhanced photocatalytic hydrogen evolution by carbon-doped carbon nitride synthesized via the assistance of cellulose. *Appl Surf Sci*. 2020; 504:144454.

37 Lv N, Li Y, Huang Z, Li T, Ye S, Dionysiou DD, Song X. Synthesis of GO/TiO_2_/Bi_2_WO_6_ nanocomposites with enhanced visible light photocatalytic degradation of ethylene. *Appl Catal B-Environ*. 2019; 246:303-311.

38 Wang J, Kumar P, Zhao H, Kibria MG, Hu JG. Polymeric carbon nitride-based photocatalysts for photoreforming of biomass derivatives. *Green Chem*. 2021; 23(19):7435-7457.

39 Wu Y, Wang F, Jin X, Zheng X, Wang Y, Wei D, Zhang Q, Feng Y, Xie Z, Chen P, Liu H, Liu G. Highly active metal-free carbon dots/g-C_3_N_4_ hollow porous nanospheres for solar-light-driven PPCPs remediation: mechanism insights, kinetics and effects of natural water matrices. *Water Res*. 2020; 172:115492.
